# The collectin SP-A and its trimeric recombinant fragment protect alveolar epithelial cells from the cytotoxic and proinflammatory effects of human cathelicidin *in vitro*

**DOI:** 10.3389/fimmu.2022.994328

**Published:** 2022-08-29

**Authors:** Lidia de Tapia, Belén García-Fojeda, Nina Kronqvist, Jan Johansson, Cristina Casals

**Affiliations:** ^1^ Department of Biochemistry and Molecular Biology, Complutense University of Madrid, Madrid, Spain; ^2^ Department of Biosciences and Nutrition, Neo, Karolinska Institutet, Huddinge, Sweden

**Keywords:** cathelicidin LL-37, collectin SP-A, trimeric recombinant fragment, antimicrobial activity, cytotoxicity, inflammation, P2X7 channel, alveolar epithelial cells

## Abstract

Human cathelicidin (LL-37) is a defense peptide with antimicrobial activity against various pathogens. However, LL-37 can also trigger tissue injury by binding to host cell membranes. The cytotoxic effects of LL-37 may be especially relevant in chronic respiratory diseases characterized by increased LL-37. The aim of this study was to investigate whether the human collectin SP-A and a trimeric recombinant fragment thereof (rfhSP-A) can regulate the activities of LL-37. To this end, we studied the interaction of LL-37 with SP-A and rfhSP-A by intrinsic fluorescence, dynamic light scattering, and circular dichroism, as well as the effects of these proteins on the antimicrobial and cytotoxic activities of LL-37. Both SP-A and rfhSP-A bound LL-37 with high affinity at physiological ionic strength (*K_D_* = 0.45 ± 0.01 nM for SP-A and 1.22 ± 0.7 nM for rfhSP-A). Such interactions result in the reduction of LL-37-induced cell permeability and IL-8 release in human pneumocytes, mediated by P2X7 channels. Binding of LL-37 to SP-A did not modify the properties of SP-A or the antibacterial activity of LL-37 against respiratory pathogens (*Klebsiella pneumoniae*, *Pseudomonas aeruginosa*, and nontypeable *Haemophilus influenzae*). SP-A/LL-37 complexes showed a greater ability to aggregate LPS vesicles than LL-37, which reduces endotoxin bioactivity. These results reveal the protective role of native SP-A in controlling LL-37 activities and suggest a potential therapeutic effect of rfhSP-A in reducing the cytotoxic and inflammatory actions of LL-37, without affecting its microbicidal activity against Gram-negative pathogens.

## 1 Introduction

Cationic host defense peptides are short amphipathic peptides (10–50 amino acids), with a net positive charge (generally +2 to +9) and antibacterial and immunomodulatory properties (1–3). There are two subfamilies of cationic defense peptides in vertebrates: cathelicidins and defensins. Defensins have a β-sheet core stabilized with disulfide bonds between six conserved cysteines, while most cathelicidins are α-helical amphipathic peptides that allow interaction and destabilization of negatively charged membranes. In humans, the only cathelicidin identified is LL-37, a 37-amino acid peptide generated from its precursor hCAP-18, which is processed extracellularly by a serin protease (e.g., neutrophil-derived proteinase 3) (1, 4, 5). LL-37 is secreted by immune and epithelial cells in the skin, intestine, ocular system, and lungs (2, 3, 5, 6). LL-37 shows low constitutive expression on skin and mucosal surfaces. However, its expression is greatly increased in response to infection, injury, inflammatory signals, and vitamin D (2, 3, 7).

LL-37 shows direct antimicrobial activity against a wide variety of microorganisms, including Gram-negative and Gram-positive bacteria, viruses, and fungi (1–3, 5), although its antimicrobial effects decrease under physiological concentrations of salt, divalent cations, host lipids, glycosaminoglycans, and lipoproteins (1–3). These observations suggest that direct antimicrobial activity of LL-37 may occur locally at sites of high cathelicidin release, since cathelicidin has a protective role in animal models of pulmonary infection by *Pseudomonas aeruginosa*, influenza virus, and *Mycobacterium bovis* BCG (1, 8). It is possible that several antimicrobial factors may act *in vivo* in combination with LL-37 since LL-37 shows cooperative action with lactoferrin (6), lysozyme (9, 10), and defensins (10, 11). On the other hand, LL-37 modulates the host immune response by chemoattracting neutrophils and eosinophils, increasing cytokine and cytokine receptor expression, inducing neutrophil and mast cell degranulation, stabilizing neutrophil extracellular traps, and modulating responses of immune cells (1–4). LL-37 also exhibits anti-inflammatory properties, especially through binding to bacterial lipopolysaccharide (LPS), which interferes with the activation of immune cells through toll-like receptors (1–3).

The antimicrobial and immunomodulatory properties of LL-37 make it a promising therapeutic agent for chronic inflammatory disorders (1) and infectious diseases, including COVID-19 (12). However, one of the limitations of using LL-37 as a therapeutic agent is its cytotoxic activity. LL-37 is capable to permeabilize host cells at high local concentrations (1, 5). LL-37 also shows proinflammatory effects, inducing the release of TNFα and IL-1β by monocytes and macrophages (3). In alveolar epithelial cells, LL-37 induces the release of the proinflammatory and chemoattractant cytokine IL-8 (13, 14). LL-37 also promotes microbe-induced apoptosis of epithelial cells while prolonging neutrophil lifespan, which could contribute to damage associated with respiratory infections (2). The deleterious proinflammatory effects of LL-37 may be especially relevant in chronic respiratory diseases, in which LL-37 is increased, such as cystic fibrosis (2, 3), chronic obstructive pulmonary disease (COPD) (2), and sarcoidosis (4). The pathogenic role of LL-37 in COPD and cystic fibrosis is inferred from the fact that LL-37 levels increase in correlation with exacerbations and symptom severity (2, 13, 15).

The host appears to protect its own cells from LL-37 cytotoxicity by several mechanisms. For example, components of human serum, including high-density lipoproteins (HDL), reduce the cytotoxicity and proinflammatory actions of LL-37 on lung epithelial cells (14), though epithelial cells generally reside in serum-free environments. The interaction of HDL apolipoprotein A-I with LL-37 reduces the cytotoxicity of LL-37 on human endothelial cells, but it also reduces the antimicrobial activity of LL-37 (16, 17). In addition, the C1q globular head receptor (gC1qR, also known as p33) protects host cells from cytolytic attack by cationic antimicrobial peptides (LL-37 and β-defensin 3) (18). p33 is a negatively charged protein expressed on the surface of various cell types. It is proposed that p33 acts as a scavenger for these cationic peptides as it binds LL-37 and β-defensin 3 with high affinity (18). In this regard, we hypothesized that the collectin SP-A, a negatively charged protein involved in pulmonary host defense, might contribute to the molecular mechanisms underlying the negative regulation of LL-37. SP-A is secreted into the airway mucosa by type II alveolar epithelial cells and non-ciliated bronchiolar cells, but it is also detected in the trachea, nasal mucosa, and other extrapulmonary mucosal surfaces, where it provides immune protection (19–21). SP-A binds to human β-defensin 3, resulting in reduced cytotoxicity on alveolar epithelial cells (22). Interestingly, LL-37 and SP-A show additive antiviral activity against influenza A virus (23), although the molecular interactions between SP-A and LL-37 remain unexplored.

**Figure 1:**
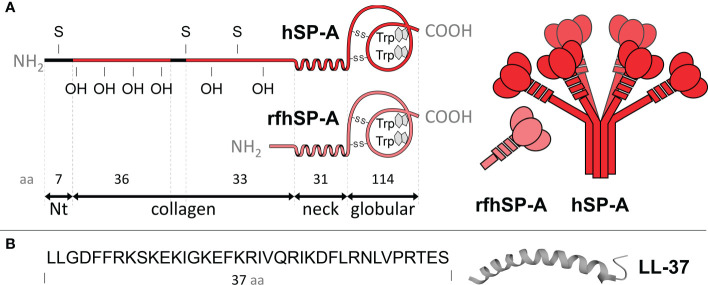
**(A)** The left panel shows the polypeptide chain domains of human SP-A and its recombinant fragment (rfhSP-A). S, denotes cysteine and OH hydroxyproline residues. The neck domain, between the collagen and globular domain, forms an α-helix structure involved in trimerization. The number of amino acids covering each domain is shown. The right panel shows schematic models of SP-A oligomers and rfhSP-A trimers. Each SP-A trimer is formed by the association of three polypeptide chains, whose collagen regions intertwine to form a collagen triple helix and the neck domains interlace to form an α-helical coiled-coil structure. A total of six trimers associate to form the characteristic “bunch of flowers” structure of SP-A. **(B)** Primary sequence of LL-37 and scheme of its α helix structure. The molecules are not drawn to scale.

SP-A constantly patrols the extracellular environment in search of pathogens, and rapidly activates several mechanisms involved in the phagocytosis of pathogens and/or bacterial killing, without inducing strong inflammatory responses (19–21, 24, 25). SP-A also contributes to the resolution of inflammation by limiting the proinflammatory activation of macrophages, promoting the removal of dead cells, and increasing the tissue repair functions of alveolar macrophages (19–21, 26, 27). SP-A has a complex oligomeric structure that facilitates its binding to a wide range of immune and non-immune ligands. Binding occurs through its N-terminal region, collagen-like region, α-helical coiled neck region, and C-terminal globular domains (19–21) (**Figure 1**). The oligomeric structure of SP-A resembles a bouquet of flowers of six trimers like the structure of mannose-binding protein (the collectin MBP) or that of the first subcomponent of the classical complement activation pathway (C1q) (21).

The complex oligomeric structure of SP-A appears to be necessary for many of its functions, as it facilitates multivalent binding to various ligands (28, 29). However, the use of small recombinant fragments of SP-A, which retain some functions of native SP-A, would have advantages in terms of ease of production and use. In this study we used a recombinant trimeric fragment of human SP-A1 (rfhSP-A), which lacks the N-terminal domain and most of the collagen domain (**Figure 1**). We produced this fragment in a novel way by using the NT* solubility tag derived from spider silk (30, 31). We previously showed that rfhSP-A is highly effective in neutralizing respiratory syncytial virus (32). The objectives of this study are to investigate whether human SP-A and rfhSP-A i) bind to LL-37 by its globular and/or neck domains and ii) modulate the antimicrobial, cytotoxic, and proinflammatory activities of LL-37. The antimicrobial activity of LL-37 was tested on three clinically relevant Gram-negative bacteria, which induce airway attacks in patients who are elderly or suffer from COPD, cystic fibrosis, or asthma.

## 2 Materials and methods

### 2.1 Materials

Human LL-37 (molecular mass, 4.5 kDa, pI: 10.6) was obtained from Sigma-Aldrich (St. Louis, MO, USA). LL-37 was resuspended in milliQ water to a final concentration of 1 mg/ml and aliquots were stored at -20°C. Rough lipopolysaccharide (Re 595, Re-LPS), from *Salmonella enterica* serotype Minnesota, and cardiolipin (CL) were obtained from Sigma-Aldrich. Palmitoyloleoyl-phosphatidylethanolamine (POPE) and palmitoyloleoylphosphatidylglycerol (POPG) were obtained from Avanti Polar Lipids (Birmingham, AL, USA). The organic solvents used to dissolve the lipids were of HPLC grade from Scharlau (Barcelona, Spain). Chocolate agar plates for the growth of nontypeable *Haemophilus influenzae* strains were from bioMérieux (Marcy l’Etoile, France). The fluorescent dye Sytox Green was from Molecular Probes (Eugene, OR, USA). Propidium podide (PI) and the P2X7 inhibitor, A-438079, were from Sigma-Aldrich. Human IL-8 ELISA kit was obtained from R&D Systems (Minneapolis, MN, USA). All other reagents were of analytical grade and were from Sigma-Aldrich.

### 2.2 Isolation of human surfactant protein A

Human SP-A was isolated from bronchoalveolar lavage fluid of patients with alveolar proteinosis using a sequential n-butanol and octylglucoside extraction as reported in (24, 25, 28, 29, 33, 34). SP-A purity was evaluated by one-dimensional SDS-PAGE in 12% acrylamide under reducing conditions and mass spectrometry. SP-A structure was analyzed by tryptophan fluorescence and circular dichroism as in (28, 29, 33). SP-A hydrodynamic diameter was determined by dynamic light scattering as in (24, 34). The degree of SP-A oligomerization was assessed by electrophoresis under nondenaturing conditions, electron microscopy, and analytical ultracentrifugation as reported elsewhere (28, 29). SP-A consisted of supratrimeric oligomers of at least 18 subunits (molecular mass, 650 kDa). Each subunit had an apparent molecular mass of 36,000 Da. Endotoxin content of isolated human SP-A was about 300 pg endotoxin/mg SP-A, as determined by Limulus amebocyte lysate assay (Pierce Biotechnology, Rockford, IL USA).

### 2.3 Expression and purification of rfhSP-A

The recombinant trimeric fragment of human SP-A (rfhSP-A) (molecular mass, 57 kDa), including the globular carbohydrate recognition domain (CRD), neck, and 8 x Gly-Xaa-Yaa repeats of the collagen stalk, was previously expressed in fusion with the wild-type NT solubility tag and purified by refolding (32). In this study, rfhSP-A was subcloned into a pT7 expression vector containing the NT* tag N-terminally of the rfhSP-A (30, 31). A His6-tag was included in the N-terminal of NT* to allow efficient purification. The cleavage site for coxsackievirus 3C protease was added between NT* and rfhSP-A to allow removal of the tag after purification. BL21 (DE3) *Escherichia coli* containing the plasmid encoding NT*-rfhSP-A were grown overnight at 37°C in LB media containing 70 mg/L kanamycin. 10 ml culture was used to inoculate 1 L of LB medium with kanamycin, and the cells were grown at 30°C to OD600 ~ 0.9. Isopropyl β-D-1-thiogalactopyranoside (IPTG) was added to a concentration of 0.5 mM, and protein was expressed for 20 h at 20°C. Cells from 1 L culture were harvested by centrifugation at 4000 x g for 20 min, and the pellet was resuspended to 60 ml in 20 mM Tris-HCl, 2 M urea, pH 8. The cell solution was sonicated (Sonics VC505 ultrasonic processor, converter model CV334, standard probe 13 mm) at 80% amplitude, 1 sec pulses, for a total of 2 min 40 sec. After lysis, a clear supernatant was obtained by centrifugation at 27,000 x g, 4°C for 30 min. The supernatant was loaded to a Ni-sepharose column (GE Healthcare) equilibrated with 20 mM Tris-HCl, 2 M urea, pH 8. The bound protein was washed with Tris buffer containing decreasing concentrations of urea (2 M, 1 M, 0.5 M and no urea) until the A280 baseline was reached. The protein was eluted with 20 mM Tris-HCl, 300 mM imidazole, pH 8, and imidazole was removed by overnight dialysis using a Spectra/Por^®^ membrane with a 6-8 kDa molecular weight cut-off placed in 5 L of 20 mM Tris-HCl, pH 8 at 4°C. The fusion protein was cleaved at 4°C overnight using 1:10 (w/w) 3C protease in the presence of 1 mM DTT. An overnight dialysis was performed as described above to remove DTT, and rfhSP-A was purified by reapplication to an IMAC column to remove His-tagged NT*. The protein was concentrated to 1.4 mg/ml using a Vivaspin 20 centrifugal tube with a 5 kDa molecular weight cut-off (GE Healthcare). rfhSP-A identity was evaluated by one-dimensional SDS-PAGE.

LPS contamination was removed by addition of polymyxin B-agarose to the rfhSP-A sample in 5 mM Tris, 150 mM NaCl, pH 7.4, at 1:5 (vol/vol). OGP (30 mM) was also added to the suspension. The sample was incubated for 30 min at room temperature in a rotator shaker and centrifuged at 500 g for 5 min at 4°C. The supernatant was then dialyzed, and the protein was quantified by the Lowry method. Endotoxin content was then determined by Limulus amebocyte lysate assay. Structural characteristics of rfhSP-A were assessed by tryptophan fluorescence and circular dichroism as in (28, 30, 33) and its hydrodynamic size by dynamic light scattering (24, 34).

The molar concentration of rfhSP-A used in most assays was six times higher than that of native SP-A, since native SP-A is composed of 6 trimeric units.

### 2.4 Spectroscopic analyses

To explore the binding between SP-A (and rfhSP-A) and LL-37 in solution the following spectroscopic analysis were performed:

#### 2.4.1 Intrinsic fluorescence

To assess the binding of human SP-A and its recombinant fragment to LL-37, the tryptophan fluorescence of rfhSP-A or SP-A was used to determine the apparent dissociation constant (K_D_) for SP-A/LL-37 and rfhSP-A/LL-37 complexes at 25°C as in (1, 5, 18, 19). Fluorescence measurements were carried out in 5 x 5 quartz cuvettes using an SLM-Aminco AB-2 spectrofluorimeter equipped with a thermostated cuvette holder (± 0.1°C; Spectronic, Waltham, MA, USA). The slit widths were 4 nm for the excitation and emission beams. The tryptophan fluorescence emission spectrum of SP-A and rfhSP-A was recorded from 305 to 400 nm on excitation at 295 nm at 25°C in 5 mM Tris HCl buffer (pH 7.4), with or without 150 mM NaCl. Subsequently, the titration experiment was started by adding increasing amounts of LL-37 to the protein solution in the cuvette. The fluorescence intensity readings were corrected for the dilution caused by LL-37 addition.

The change in the fluorescence of SP-A (or rfhSP-A) at the emission wavelength maximum was monitored as a function of LL-37 concentration, and the titration data were analyzed by nonlinear least squares fitting to the Hill equation, as previously reported (24, 35, 36):


(1) $$\Delta F/\Delta F_{\max}={\left[L\right]}^{nH}/({\left[L\right]}^{nH}+K_{D})$$

where ΔF is the change in fluorescence intensity at 337 nm relative to the intensity of free SP-A (for free rfhSP-A, the emission wavelength maximum was 342 nm); ΔFmax is the change in fluorescence intensity at saturating LL-37 concentrations; K_D_ is the apparent equilibrium dissociation constant; [L] is the molar concentration of free LL-37; and nH is the Hill coefficient.

#### 2.4.2 Dynamic light scattering (DLS)

Hydrodynamic diameters of LL-37, SP-A, rfhSP-A, and combinations thereof were measured at 25°C in a Zetasizer Nano S (Malvern Instruments, Malvern, UK) equipped with a 633 nm HeNe laser as in (24, 34–37). Interaction of SP-A or rfhSP-A with LL-37 in solution was measured by addition of different concentrations of LL-37 to 15 nM SP-A (or rfhSP-A) in 5 mM Tris-HCl buffer (pH 7.4), in the absence or presence of 150 mM NaCl. Six scans were performed for each sample, and experiments were performed in triplicate. The hydrodynamic diameters were calculated using the general purpose and multiple narrow modes algorithms available from the Malvern software for DLS analysis.

#### 2.4.3 Circular dichroism measurements

Circular dichroism (CD) spectra were obtained on a Jasco J-715 spectropolarimeter fitted with a 150 W xenon lamp (38). Quartz cells with a 0.1 cm optical path length were used, and the spectra were recorded in the far-UV region (190-260 nm) with a scanning speed of 50 nm/minute at 25°C. Four scans were accumulated and averaged for each spectrum. The acquired spectra were corrected by subtracting the appropriate blanks, subjected to noise reduction analysis, and presented as molar ellipticities (degrees cm2 dmol-1) assuming 110 Da as the average molecular mass per amino acid residue. At least three independent preparations of LL-37, SP-A, and rfhSP-A were measured. Spectra Manager Software (Jasco version 1.53) was used.

The dichroism spectra of LL-37 in the presence or absence of SP-A, rfhSP-A, and/or 1 mg/ml lipid vesicles were measured after thermostatization of the samples for 10 minutes. Two types of lipid vesicles were used: vesicles mimicking the bacterial cell wall (Re-LPS/BPL at a ratio of 08:02 by weight), and vesicles of bacterial inner membrane phospholipids (BPL). The final concentrations were 100 μg/ml (22.2 μM) for LL-37, 75 μg/ml (0.11 μM) for SP-A, and 75 μg/ml (1.3 μM) for rfhSP-A, in a final volume of 200 μl of 5 mM Tris-HCl, NaCl 150 mM, pH 7.4 buffer.

### 2.5 SP-A self-association assay

Self-association assays of SP-A were performed as previously described (38, 39) by measuring the Ca^2+^ dependent change in protein absorbance at 360 nm in a DU-800 spectrophotometer (Beckman Coulter, Fullerton, CA, USA) at 37°C.

### 2.6 Preparation of bacterial lipid vesicles and lung surfactant-like vesicles

Vesicles mimicking the inner membrane of Gram-negative bacteria were prepared by mixing POPE, POPG, and CL at weight ratios of 67:23:10. Alternatively, vesicles mimicking bacterial outer membrane were prepared by adding ReLPS to the POPE/POPG/CL mixture (BPL, bacterial phospholipids) at a weight ratio of 8:2, as previously reported (25). Multilamellar vesicles and large unilamellar vesicles of DPPC and surfactant-like vesicles composed of DPPC, POPG, and palmitic acid (PA) (at weight ratios of 23:10:1.6) were prepared as previously reported (28, 33–35, 39).

### 2.7 LPS vesicle aggregation assays

Turbidity measurements: The effects of SP-A, rfhSP-A, and calcium on the aggregation of LPS vesicles that mimic bacterial outer membranes were studied in the absence and presence of LL-37 by measuring the change in absorbance at 400 nm in a Beckman DU-800 spectrophotometer (28, 39). Briefly, LPS vesicles (50 μg/ml) were added to both the sample and the reference cuvettes in 5 mM Tris-HCl and 150 mM NaCl 0.1 mM EDTA buffer, pH 7.4. After 10 min equilibration at 37°C, either human SP-A, rfhSP-A, LL-37, or combinations thereof was added to the sample cuvette, and the change in optical density at 400 nm was monitored. Next, 2.5 mM Ca^2+^ was added to both the sample and reference cuvettes, and the change in absorbance was monitored again. Aggregation of surfactant phospholipid vesicles was performed in the same way.

Dynamic Light Scattering (DLS): Samples used for turbidity measurements in the presence of calcium were also analyzed by DLS to determine the protein effect on the intensity-based size distribution and the hydrodynamic diameter (Z-average) of lipid/protein material as in (36).

### 2.8 Bacterial strains, media, and growth conditions

*Klebsiella pneumoniae* 52145 (serotype K2:O1), nontypeable *Haemophilus influenzae* strain 375 (NTHi), and *P. aeruginosa* (PAO1) are clinical isolates, as previously described (40–42). *P. aeruginosa*, and *K. pneumoniae* K2 were grown in Luria–Bertani (LB) broth at 37°C with continuous shaking to the exponential phase. Frozen stocks of NTHi strains were thawed and then grown on chocolate agar plates during 18 h at 37°C in a humidified 5% CO2 atmosphere. Then, NTHi were grown to the exponential phase on brain heart infusion broth (BHI) supplemented with 10 µg/ml hemin and 10 µg/ml β-nicotinamide adenine dinucleotide (β-NAD) (sBHI) with continuous shaking at 37°C in a humidified 5% CO^2^ atmosphere. Exponential-phase bacteria were then harvested by centrifugation at 500 g for 10 min, resuspended in PBS, and adjusted to the desired final concentration, as described in (24).

### 2.9 Bacterial killing assays

The microbicidal activity of LL-37 in the presence of SP-A or rfhSP-A was evaluated by colony counts on plate assays (24, 25). Five microliters of bacterial suspension in the exponential phase were incubated with different concentrations of LL-37 in the presence or absence of SP-A or rfhSP-A in a final volume of 30 µl of phosphate buffered saline (PBS) composed of 137 mM NaCl, 2.7 mM KCl, 6.5 mM Na_2_HPO_4_, 1.47 mM KH_2_PO_4_, pH 7.4 for 1 h at 37°C. For NTHi killing assays, PBS contains 1% of trypticase soy broth (TSB). In all cases the final bacterial concentration was 10^5^ CFU/ml. At the end of incubation, bacterial suspensions were plated in LB agar for *K. pneumoniae* and PAO1, or sBHI agar for NTHi, and incubated for 18 h at 37°C. Viable bacteria were enumerated by colony count and results were expressed as a percentage of relative survival in comparison to untreated bacteria.

### 2.10 Bacterial membrane permeabilization

The ability of LL-37, SP-A, rfhSP-A, and combinations thereof to permeabilize the outer and cytoplasmic bacterial membranes was studied in live bacteria by quantifying the internalization of the impermeant fluorescent Sytox Green, since its fluorescence increases when binding to bacterial DNA. For the measurement of Sytox Green influx, the probe (1 μM) was added to 1 ml of bacterial suspension (2x10^7^ CFU/ml) in PBS and the sample was incubated for 15 min in darkness at room temperature as described in (25). Then, the fluorescence of the Sytox Green/bacterial suspension mixture was monitored for 4 hours in a FLUOstar Omega microplate reader (BMG LabTechnologies, Ortenberg, Germany) at excitation and emission wavelengths of 485 and 520 nm, respectively. PBS was used as a negative control, whereas ethanol (70%) was used as a positive control. Background fluorescence was measured in non-labeled bacteria.

### 2.11 Transmission electron microscopy

The effect of LL-37, SP-A, rfhSP-A, and combinations thereof on the ultrastructure of *K. pneumoniae* was visualized by means of transmission electron microscopy, as described in (25). *K. pneumoniae* bacteria in the mid-logarithmic growth phase (2 x 10^8^ CFU/ml) were treated with 55.5 LL-37, of 0.3 µM SP-A, 1.75 µM rfhSP-A SP-A, mixed LL-37/SP-A and mixed LL-37/rfhSP-A at 37°C for 30 min in PBS buffer. Cells were spun down and PBS medium was removed. Cell pellets were then chemically fixed with 4% paraformaldehyde and 2.5% glutaraldehyde for 4 h at 4°C and washed three times with PBS. Next, bacteria were post-fixed with 1% osmium tetroxide for 1 h. Samples were then washed thrice with bi-distilled water and dehydrated using sequential exposure to acetone concentrations ranging from 30% to 100% for 15 min at room temperature. Next, infiltration and embedding were performed using Spurr’s resin. The samples were sectioned using an ultramicrotome with a diamond knife and were mounted on copper grids. Samples were examined on a JEOL JEM 1010 electron microscope (JEOL, Tokyo, Japan).

### 2.12 Cell assays

Human cell lines of alveolar basal epithelium A549 (ATTC^®^ CCL-185TM) and monocytes U937 (ATTC^®^ CRL-1593.2TM) were incubated in RPMI 1640 supplemented with 10% (v/v) heat-inactivated fetal bovine serum (FBS), antibiotics (100 U/ml penicillin and 100 μg/ml streptomycin), and 2 mM L-glutamine (BioWhittacker). Cells were maintained at 37°C in a humidified 5% CO2 atmosphere and used to analyze the cytotoxic and inflammatory activity of LL-37.

#### 2.12.1 Sytox Green incorporation

Cytotoxic activity of LL-37 was evaluated in the presence or absence of SP-A or rfhSP-A by quantifying the internalization of the impermeant fluorescent Sytox Green, since its fluorescence increases when binding to DNA. Incorporation of Sytox Green in A549 cells was analyzed by confocal microscopy and flow cytometry analysis.

For confocal microscopy analysis, cells were seeded to a density of 30,000 cells per well in a 24-well tissue culture plate for 18 h in RPMI 1640 tissue culture medium supplemented with 100 U/ml penicillin, 100 μg/ml streptomycin, 2 mM L-glutamine, and with 5% FBS. The following day, Sytox Green (1 µM, final concentration) was added to the wells 10 minutes before the addition of 2.5 µM (11.25 µg/ml) LL-37 and/or 0.15 µM (100 µg/ml) SP-A or 0.88 µM (50 µg/ml) rfhSP-A for an additional 30 minutes. At the end of the incubation, fluorescence and differential interference contrast micrographs were taken under an Olympus FV1200 confocal system. Quantification of permeabilized cells (Sytox Green+) per cell number was performed using Image J software on each of 12 micrographs per treatment and experiment.

For flow cytometry analysis, A549 cells were grown in RPMI 1640 medium supplemented with antibiotics, 2 mM L-glutamine, and 10% FBS. Cells were harvested and incubated in 300 µl (final volume) PBS buffer at a concentration of 267,000 cells/ml with 1 µM Sytox Green, 2.5 µM LL-37, in the presence or absence of 0.15 µM SP-A or 0.88 µM rfhSP-A. In some experiments, cells were pre-treated 15 minutes with 10 µM A-438079, a P2X7 specific inhibitor (43) or DMSO. A-438079 stock was resuspended in DMSO to a final concentration of 30 mM and aliquots were stored at 4°C. Cell fluorescence was analyzed after 15, 30 and 60 minutes in a Becton-Dickinson FACS Calibur cytometer, using Cell Quest software.

#### 2.12.2 Propidium iodide uptake by human cells

The incorporation of PI in A549 and U937 cell lines was analyzed by flow cytometry. Cells were cultured 18 h in RPMI 1640 medium supplemented with antibiotics, 2 mM L-glutamine, and 10% FBS. Then, cells were harvested and incubated in PBS with 10 µg/ml PI in the presence or absence of 2.5 µM LL-37, 0.15 µM SP-A or 0.88 µM rfhSP-A, and combinations thereof. A549 were incubated at 500,000 cells/ml density and U937 at 1,000,000 cells/ml. PI incorporation was measured after 15, 30, 45 and 60 minutes by flow cytometry. In some experiments, cells were pre-treated with 1 or 10 µM A-438079 or vehicle (DMSO). Then, cells were exposed one hour to 2.5 µM LL-37 in the presence or absence of 0.15 µM (100 µg/ml) SP-A or 0.88 µM (50 µg/ml) rfhSP-A, and PI uptake was analyzed in a Becton-Dickinson FACS Calibur cytometer, using Cell Quest software.

#### 2.12.3 LL-37-induced IL8 secretion

A549 cells were seeded to a density of 50,000 cells per well in a 96-well tissue culture plate for 18 h in RPMI 1640 medium supplemented with antibiotics, 2 mM L-glutamine, and 5% FBS. The following day, cells were exposed 24 h to 15 μM LL-37 in the presence or absence of 0.15 μM SP-A or 0.52 μM rfhSP-A. In some experiments, cells were pre-treated 15 minutes with 10 µM A-438079 to inhibit P2X7 activation or DMSO.

Secreted IL-8 was quantified in supernatants of A549 cells by ELISA (R&D Systems) following the supplier instructions. Briefly, anti-human IL-8 was coated on a 96-well Nunc-Immuno Plate MaxiSorp Surface (Thermo Scientific, Waltham, MA) in PBS overnight. After blocking with PBS and 10% FBS, and extensive washing, samples and standards were incubated for 2 h at room temperature. IL-8 cytokine was detected with biotinylated detection antibody and streptavidin-HRP. The colorimetric reaction was developed with tetramethylbenzidine (BD Biosciences, San Diego, CA) and was stopped with 4 M sulfuric acid. The absorbance at 450 nm was read on an ELISA reader (RT-6100 plate reader, Rayto Life and Analytical Sciences, Shenzhen, China).

### 2.13 Statistical analysis

Data are presented as means ± SD. Differences in the means were analyzed by one-way ANOVA followed by the Bonferroni multiple-comparison test. For comparison of two groups, Student t test was used. An α level ≤ 5% (p ≤ 0.05) was considered significant.

## 3 Results

### 3.1 Binding of LL-37 to SP-A and rfhSP-A and formation of molecular aggregates

The potential interaction between SP-A and rfhSP-A with LL-37 was evaluated by quantifying the apparent dissociation constant, *K_D_*, and the aggregation state of the LL-37/SP-A or LL-37/rfhSP-A mixtures in solution. These studies were performed in the absence and presence of salts to further determine the role of NaCl in the interaction between positively charged LL-37 (pI:10.6) and negatively charged SP-A or its fragment, whose isoelectric point varies between pH 4.9 and 5.2 (44).

**Figure 2:**
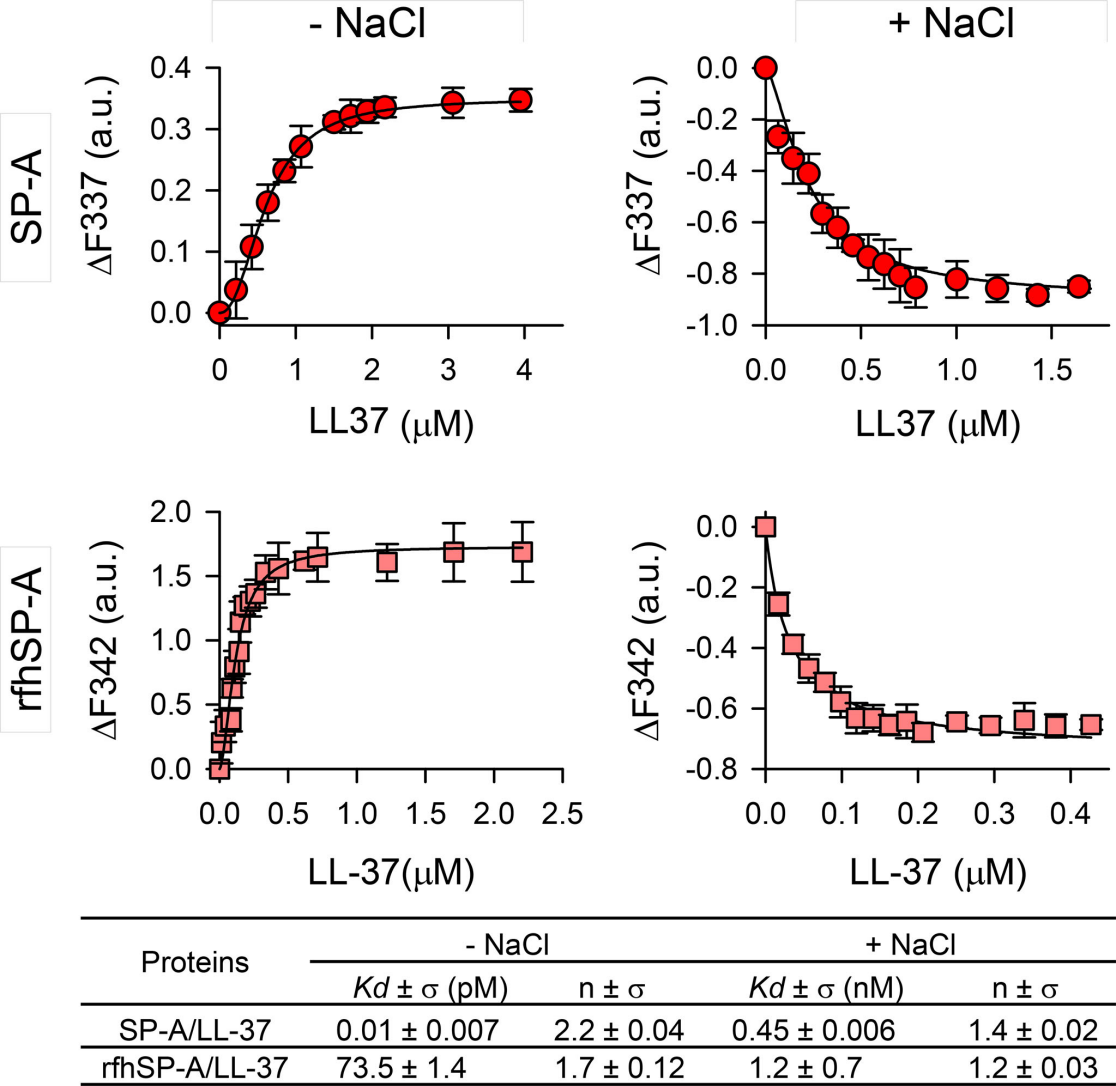
SP-A and rfhSP-A interact with LL-37 in a dose-dependent manner. Tryptophan fluorescence emission spectra of SP-A (75 nM) and rfhSP-A (52 nM) were measured with increasing concentrations of LL-37 at 25°C in 5 mM Tris-HCl buffer (pH 7.4) with (right panels) and without (left panels) 150 mM NaCl. SP-A or rfhSP-A samples (with and without LL-37) and blank samples (LL-37 alone) were excited at 295 nm and the emission spectra recorded from 300 to 450 nm. Results are expressed as ΔF of the corrected emission intensity at the emission maximum wavelength. Results are means ± SD of three independent experiments. Apparent *K_D_* values and Hill coefficients for the binding of SP-A and rfhSP-A to LL-37 in the presence and absence of physiological concentrations of NaCl are shown.

**Figure 2** shows the binding of LL-37 to SP-A and rfhSP-A by following the change in SP-A tryptophan fluorescence. The fluorescence of SP-A or rfhSP-A is dominated by the contribution of two conserved tryptophan residues at the globular domains of these proteins (33), while LL-37 lacks tryptophan residues. Titration of SP-A or rfhSP-A with LL-37 in the absence of NaCl increased the protein’s intrinsic fluorescence in a dose-dependent manner (**Figure 2**, left panels), indicating that SP-A and rfhSP-A bind to LL-37. Fitting the titration data at the emission maximum wavelength to the Hill equation yielded *K_D_* values of 0.01 ± 0.007 pM for SP-A/LL-37 interaction and 73.5 ± 1.4 pM for rfhSP-A/LL-37. The Hill coefficients, nH, obtained (2.2 ± 0.04 for SP-A/LL-37 and 1.7 ± 0.12 for rfhSP-A/LL-37) are indicative of cooperative binding.

The binding affinity was reduced in the presence of NaCl (**Figure 2**, right panels). Addition of increasing concentrations of LL-37 resulted in a significant LL-37 concentration-dependent decrease in the amplitude of the fluorescence emission spectrum of SP-A or rfhSP-A, without any shift in the wavelength of the emission maximum. The decrease in protein fluorescence is a consequence of conformational changes in these proteins induced by salts, as previously reported (38, 45), which increase with increasing LL-37 concentration. The estimated *K_D_* for SP-A/LL-37 and rfhSP-A/LL-37 interactions were 0.45 ± 0.006 nM and 1.2 ± 0.7 nM, respectively, and the Hill coefficient values were greater than 1, indicating a positive cooperative binding.

**Figure 3:**
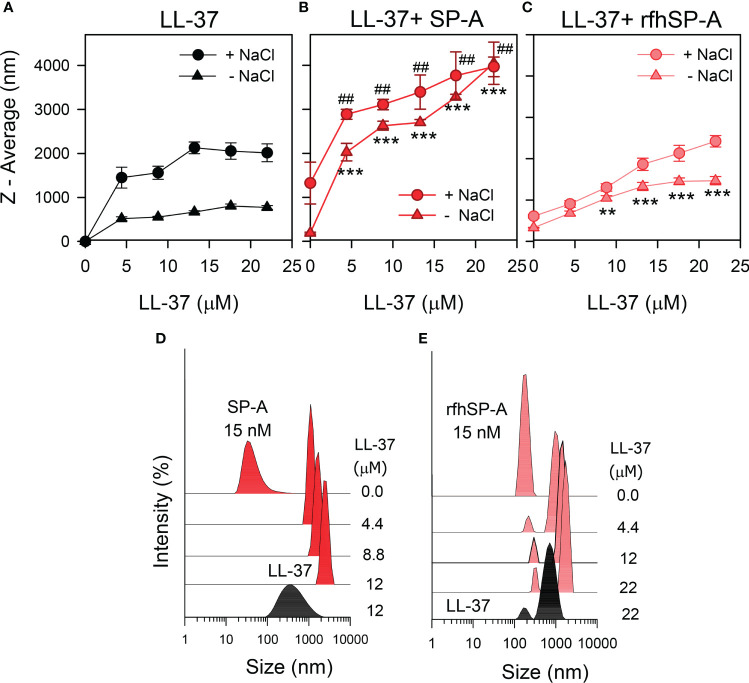
SP-A and rfhSP-A form molecular aggregates with LL-37. The formation of complexes between LL-37 and either SP-A or rfhSP-A in solution was examined in the presence and absence of salts by dynamic light scattering. **(A)** NaCl effect on the overall Z-average of LL-37 particles. **(B)** Dependence of Z-average of LL-37/SP-A mixtures on the concentration of LL-37 in the presence and absence of NaCl. **(C)** Dependence of Z-average of LL-37/rfhSP-A mixtures on the LL-37 concentration, with and without NaCl. SP-A and rfhSP-A concentrations in **(B, C)** were 10 µg/ml for SP-A and 0.87 µg/ml for rfhSP-A, corresponding to 15 nM. Results are the mean ± SD of four experiments. For statistical analysis, Student’s T-test was used: **p < 0.01; ***p < 0.001 when the effect of the presence of SP-A or rfhSP-A in the mixture was compared with LL-37 alone in the absence of NaCl. ^##^ *p* < 0.001when the effect of the presence of SP-A or rfhSP-A in the mixture was compared with LL-37 alone in the presence of NaCl. In **(D)** it is shown a representative experiment of the formation of SP-A/LL-37 aggregates upon addition of increasing concentration of LL-37 to a solution containing a constant concentration of SP-A (15 nM) in 5 mM Tris-HCl buffer (pH 7.4). DLS analyses of LL-37 particles alone is also shown in the graph. In **(E)** a representative experiment of the formation of rfhSP-A/LL-37 aggregates is also shown under the same conditions as in **(D)** In **(D, E)**, the y-axis is the relative intensity of the scattered light, and the x-axis represents the hydrodynamic diameter of the particles present in the solution.

To determine whether there are changes in the size of LL-37 particles after interaction with SP-A or rfhSP-A in solution, we performed dynamic light scattering in the absence and presence of salts (**Figure 3**). **Figure 3A** shows that the presence of NaCl affected particle size of LL-37, which increased with increasing LL-37 concentration. This is consistent with the expected behavior of an amphiphilic α-helix such as LL-37 in which monomeric α-helical LL-37 is in equilibrium with an α-helical oligomer form in the presence of physiological concentrations of salts (46, 47). **Figure 3B** shows that addition of LL-37 to a solution containing SP-A, at neutral pH, caused a LL-37 concentration-dependent increase of SP-A hydrodynamic size, both in the presence and absence of salts, as a consequence of the formation of LL-37/SPA aggregates (**Figures 3B, D**). Thus, our results show that the addition of LL-37 to SP-A in solution caused the disappearance of SP-A and LL-37 peaks and the appearance of a new peak, which presumably consists of LL-37/SP-A aggregates of greater hydrodynamic size than that determined for LL-37 and SP-A particles alone. On the other hand, addition of LL-37 to a solution containing rfhSP-A resulted in the formation of LL-37/rfhSPA aggregates, which were significantly greater that those formed by LL-37 alone in the absence of salts (**Figures 3C, E**).

### 3.2 Secondary structure of LL-37, SP-A, rfhSP-A, and combinations thereof in the presence and absence of bacterial lipids

CD spectroscopy in the far UV region (190–260 nm) is a powerful technique to probe conformational changes that occur during protein-protein interactions in solution (48). In addition, CD spectroscopy is important to check the content of α-helical structure of LL-37 in the absence and presence of SP-A and rfhSPA, because optimal antibacterial activity of LL-37 requires an α-helical structure (46) and low antibacterial activities of LL-37 correspond to decreased helical content and increased disordered structure (46, 47). Thus, we investigated whether interactions between LL-37 and SP-A or its trimeric fragment result in a change in LL-37 conformation.

**Figure 4:**
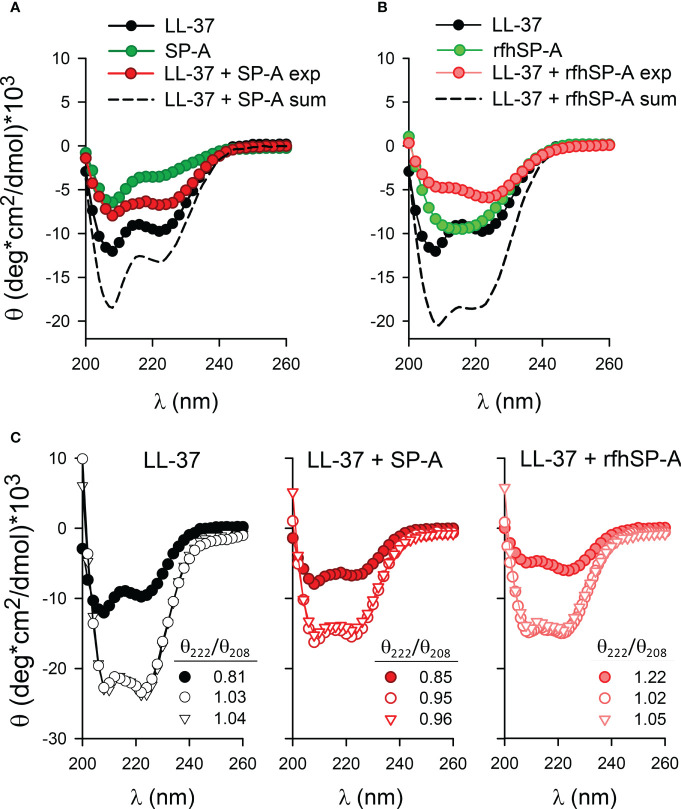
Secondary structure of LL-37, SP-A, rfhSP-A, and combinations thereof in the presence and absence of bacterial membranes. **(A)** The mean residue ellipticity of LL-37, SP-A, the mixture of LL-37 and SP-A proteins (denoted exp), and the sum of the individual spectrum of each protein (dashed line, denoted sum). **(B)** The mean residue ellipticity of LL-37, rfhSP-A, the mixture of LL-37and rfhSP-A proteins (denoted exp), and the sum of the individual spectrum of each protein (dashed line, denoted sum). **(C)** Changes in the molar ellipticity of LL-37, LL37+SP-A, and LL-37+rfhSP-A in the absence (filled circles) and presence of lipid vesicles mimicking the bacterial inner membrane (open triangles) (BPL: POPE/POPG/CL, 67/23/10, w/w/w, 1 mg/ml) and the outer membrane of Gram-negative bacteria (open circles) composed of Re-LPS/BPL (8:2, w/w) (1 mg/ml). Mean residue 222/208 ellipticity ratios are shown. Measurements were performed at 25°C in 5mM Tris-HCl, 150mM NaCl buffer, pH 7.4. Protein concentrations were: 100 µg/ml LL-37, 75 µg/ml SP-A, and 75 µg/ml rfhSP-A. A representative experiment of three are shown.

**Figures 4A, B** show that, as expected, the CD spectrum of LL-37 recorded in 5mM Tris-HCl, 150 mM NaCl buffer, pH 7.4, revealed an α-helical signature with minima at 208 and 222 nm. The circular dichroic spectra of human SP-A in 5mM Tris-HCl, pH 7.4, is characterized by a strong negative extreme at 207 nm and a shoulder at 223 nm, as previously reported (38, 45, 49, 50). In the presence of 150 mM NaCl, circular dichroic spectra of SP-A show a marked decrease of negative ellipticity without any shift of the minimum at 207 nm, as a consequence of self-aggregation of the protein in the presence of salts (38). To assess whether LL-37 conformation changed upon LL-37/SP-A interaction, we coincubated LL-37 and SP-A in 5mM Tris-HCl, 150mM NaCl buffer, pH 7.4, and their CD spectra were recorded between 195 and 260 nm. The CD spectrum of the protein mixture (LL-37+SP-A) was compared with the sum of the individual spectra of each interacting partner (**Figure 4A**). The experimental and calculated spectra of LL-37+SP-A mixtures differ, showing a marked loss in α-helical content in the experimental LL-37+SP-A spectra. The decrease in the dichroic signal of the protein mixture might be associated with the formation of molecular aggregates of LL-37/SP-A. Moreover, **Figure 4B** shows the CD spectrum of the LL-37 + rfhSP-A mixture compared with the sum of the individual spectra of each interacting protein. Again, the experimental and calculated spectra of LL-37 + rfhSP-A mixtures differ, showing a clear loss in α-helical content in the experimental LL-37+rfhSP-A spectra. The decrease in the dichroic signal is consistent with the formation of molecular aggregates of LL-37/rfhSP-A.

To investigate the impact of the binding of these proteins to bacterial model membranes, we prepared lipid vesicles that mimic the inner membrane (denoted BPL and composed of POPE/POPG/CL at ratios of 67:23:10 by weight) and the outer membrane (ReLPS/BPL at a weight ratio of 8:2) of Gram-negative bacteria (25). The secondary structures of LL-37 and LL-37/SP-A or LL-37/rfhSP-A complexes were determined by CD in the presence and absence of LUVs of Re-LPS/BPL and BPL (**Figure 4C**). The % of α-helical content was determined by using the K2D3 tool (51). **Figure 4C** shows that LL-37 exhibited a large increase in helicity through interaction with both Re-LPS/BPL and BPL vesicles at LL-37/lipid molar ratios of 1/15 and 1/55, respectively. The α-helical content increased from around 17% to 80% upon interaction with bacterial membranes. The θ_222_/θ_208_ ratio for LL-37 increased upon interaction with LUVs composed of Re-LPS/BPL or BPL (from 0.81 to 1.03 and 1.04, respectively), and it was determined empirically that the θ_222_/θ_208_ ratio is equal to or greater than 1 for two-stranded coiled-coils, whereas, for non-interacting helices, the ratio is less than 0.86 (52). **Figure 4C** also shows that the α-helical content of LL-37/SP-A or LL-37/rfhSP-A complexes markedly increased after interaction with bacterial model membranes (from around 4% to 38%). The θ_222_/θ_208_ ratio for LL-37/SP-A increased upon interaction with bacterial membranes, from 0.85 to 0.95 (Re-LPS/BPL) and to 0.96 (BPL), whereas the θ_222_/θ_208_ ratio for LL-37/rfhSP-A slightly decreased from 1.22 to 1.02 (Re-LPS/BPL) and 1.05 (BPL). Together, these data indicate that LL-37/SP-A and LL-37/rfhSP-A complexes bind to bacterial membranes and maintain an optimal α-helical structure required for LL-37 antibacterial activity.

### 3.3 Effects of LL-37 on Ca^2+^-dependent properties of SP-A

**Figure 5:**
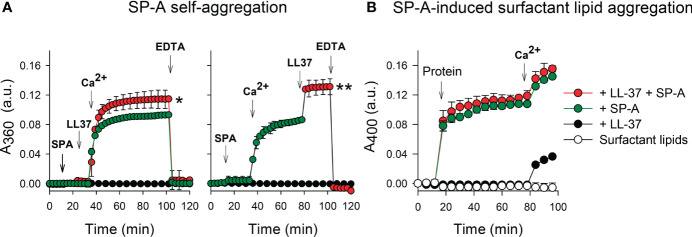
Effects of LL-37 on Ca^2+^-dependent properties of SP-A. **(A)** The effect of LL-37 on the kinetic of self-aggregation of SP-A was determined by measuring the change in turbidity at 360 nm. The sample and reference cuvettes were filled with 5 mM Tris-HCl buffer (pH 7.4), and after 10 min equilibration at 37°C, SP-A (75 nM, 50 µg/ml) was added. The absorbance change at 360 nm was monitored at 37°C at 1 min intervals. LL-37 (1.1 µM) was added to the solution containing SP-A (o buffer) before (left) or after (right) addition of 5 mM Ca^2+^ (final concentration). Self-aggregation was reverted by EDTA (10 mM, final concentration). **(B)** The effect of LL-37 on surfactant lipid aggregation induced by SP-A was determined by measuring the change in turbidity at 400 nm. Sample and reference cuvettes were first filled with 80 μg/ml surfactant-like vesicles composed of DPPC/POPG/PA (23:10:1.6, w/w/w) in 5 mM Tris-HCl buffer, pH 7.4, containing 150 mM NaCl. After a 10-min equilibration at 37°C, SP-A (50 μg/mL), LL-37 (1.1 µM), or SP-A+ LL-37 were added to the sample cuvette, and the change in absorbance at 400 nm was monitored. Next, CaCl_2_ (2.5 mM) was added to both sample and reference cuvettes. Control experiments were performed adding buffer instead of proteins to the lipid vesicle suspension in the cuvette. In **(A, B)**, results are shown as means ± SD of three independent experiments. For statistical analysis, Student’s T-test was used. *p < 0.05 and **p < 0.01 when self-aggregation of SP-A+LL-37 was compared with that of SP-A.

Given that calcium is present in the alveolar fluid at a concentration of 2 mM and that SP-A binds calcium, modifying SP-A conformation and its state of self-association (38, 39, 44), we studied the effect of physiological concentrations of calcium on the colloidal stability of SP-A/LL-37 mixtures. **Figures 5A** show that the binding of LL-37 to SP-A, before (left) or after (right) addition of calcium, significantly increased Ca^2+^-dependent self-association of SP-A, measured by change of absorbance at 360 nm. Ca^2+^-dependent SP-A/LL-37 aggregates were dissociated by EDTA. The trimeric fragment of SP-A could not undergo Ca^2+^-dependent self-association either in the presence or absence of LL-37 (**Supplementary Figure 1**), which is consistent with the fact that self-association of SP-A in the presence of calcium requires the protein to be in a supratrimeric assembly and full trimers of SP-A are unable to self-associate (29).

**Figure 5B** shows that the presence of LL-37 in SP-A/LL-37 complexes did not affect the capability of SP-A to aggregate surfactant-like vesicles composed of DPPC, POPG, and palmitic acid (23:10:1.6, w/w/w). This process predicts the surface-active properties of SP-A in concerted action with surfactant protein SP-B (44, 53). LL-37 binds to zwitterionic DPPC (47, 54), but in contrast to SP-A, LL-37 was unable to induce Ca^2+^-dependent DPPC vesicle aggregation. However, SP-A/LL-37 complexes can induce DPPC membrane aggregation in the presence of calcium (**Supplementary Figure 2**). Together these results indicate that the binding of LL-37 to native SP-A does not interfere on the two Ca^2+^ dependent related phenomena in which self-associated SP-A molecules connect surfactant membranes by interaction of their globular heads with membrane surfaces of contiguous bilayers, a special feature that contributes to the structure and function of pulmonary surfactant (44, 53).

### 3.4 Ca^2+^-dependent aggregation of LPS vesicles

**Figure 6:**
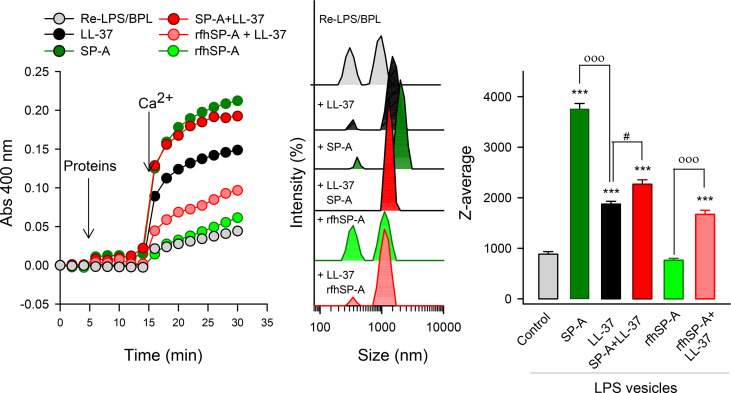
Ca^2+^-dependent aggregation of LPS vesicles induced by SP-A, LL-37, rfhSP-A, and combinations thereof. Vesicles mimicking Gram-negative bacterial outer membrane composed of Re-LPS/BPL (8:2, w/w) (50 μg/ml) in 5 mM Tris-HCl buffer, pH 7.4, containing 150 mM NaCl and 0.1 mM EDTA were used. The left panel represent one representative experiment of three of LPS vesicle aggregation measured by turbidity at 400 nm. The central panel shows the samples used for turbidity measurements analyzed by DLS. The right panel shows the Z-average measurements of these samples. Results are the mean ± SD of three experiments. For statistical analysis, ANOVA followed by Bonferroni multiple comparison test was used. ***p < 0.001 when protein-treated samples are compared with the control (LPS vesicles). ^ooo^p < 0.001 when samples with SP-A/LL-37 or rfhSP-A/LL-37 are compared with SP-A- or rfhSPA-samples, respectively. ^#^p < 0.05 when SP-A/LL-37- and LL-37-samples are compared. The concentrations of proteins were 75 nM SP-A, 1.1 µM LL-37, and 0.43 µM rfhSP-A. The concentration of Ca^2+^ was 2.5 mM.

Next, we determined the capability of LL-37, SP-A, rfhSP-A, and combinations thereof to aggregate LPS vesicles that mimic the outer membrane of Gram-negative bacteria (ReLPS/BPL at a weight ratio of 8:2) (**Figure 6**). Both LL-37 and SP-A are LPS binding proteins (1, 3, 20, 21, 28, 29, 55). Aggregation of LPS-containing membranes is important to neutralize bacterial lipopolysaccharide, an endotoxin that causes severe hyperinflammation and pathology. **Figure 6** (left panel) shows that addition of either SP-A, LL-37, rfhSP-A, or combinations thereof to LPS vesicles did not affect sample turbidity. However, in the presence of 2.5 mM calcium, the amount of scattered light induced by either SP-A, LL-37, or complexes of SP-A/LL-37 and rfhSP-A/LL-37, but not rfhSP-A alone, greatly increased.

To further characterize the effect of calcium on the aggregation state of protein/LPS vesicles, samples used for turbidity measurements were analyzed by DLS (**Figure 6**, central and right panels). Turbidity measurements and DLS analyses indicated that LL-37 induced LPS vesicle aggregation in the presence of Ca^2+^, although the magnitude of this process is significantly smaller than that induced by SP-A alone. Interestingly, the ability of SP-A/LL-37 complexes to aggregate LPS vesicles was significantly greater than that of LL-37 alone. On the other hand, the trimeric recombinant fragment of SP-A cannot agglomerate LPS vesicles in the presence of calcium, despite binding to these membranes (**Supplementary Figure 3**). Importantly, rfhSP-A/LL-37 complexes maintain the ability of LL-37 to aggregate LPS vesicles.

### 3.5 Antimicrobial activity of SP-A/LL-37 and rfhSP-A/LL-37 complexes against respiratory pathogens

**Figure 7:**
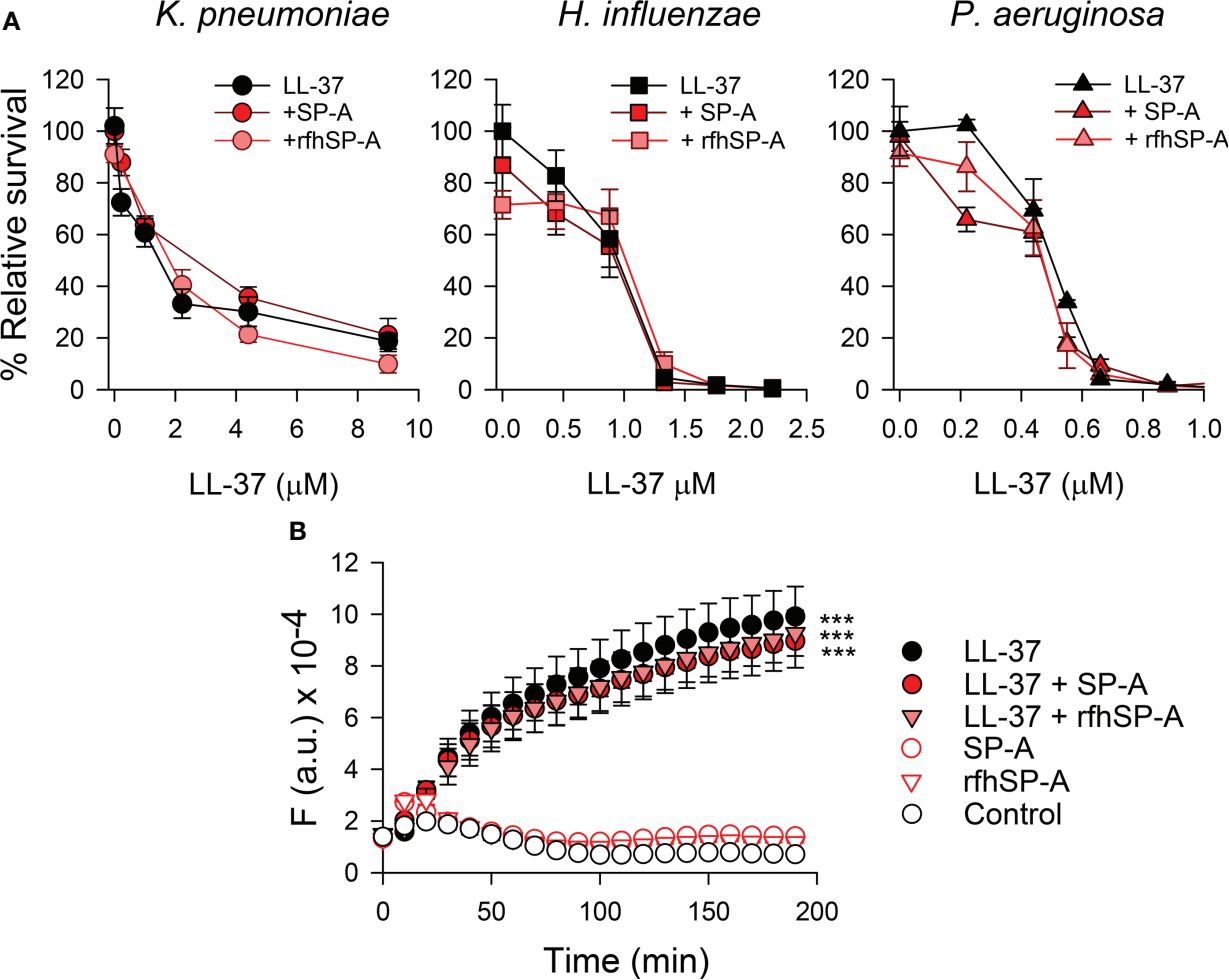
**(A)** Antimicrobial activity of LL-37 against *K pneumoniae*, non-typable *H influenzae*, and *P. aeruginosa*, in the presence and absence of SP-A or rfhSP-A. 10^5^ CFUs/ml of *K pneumoniae* K2, NTHi, and *P. aeruginosa* O1 were incubated with different concentrations of LL-37 in the absence and presence of SP-A and rfhSP-A in PBS (pH 7.4), for 1 h at 37°C. The final concentration of SP-A was within the ranges found in the alveolar fluid of human lungs: 0.11 µM for the *K pneumoniae* assay, 0.025 µM for the NTHi assay, and 0.012 µM for the *P. aeruginosa* assay. These concentrations were used to maintain a similar molar ratio between LL-37 and SP-A in each killing assay. Regarding rfhSP-A, the final molar concentration in each assay was six times higher than that of SP-A (0.7 µM, 0.15 µM, and 0.075 µM, respectively) since native SP-A is composed of 6 trimeric units. Bacteria were then plated on LB agar (*K. pneumoniae* and PAO1) or sBHI agar (NTHi) for CFU counting. Results are shown as a percentage of relative survival compared to untreated bacteria. Data are means ± S.E.M. of three independent experiments, with three biological replicates. **(B)** Membrane integrity of Gram-negative bacteria. 10^7^ CFU/ml of *K pneumoniae* were incubated with 2.2 µM LL-37 and/or (0.075 µM) SP-A and (0.4 µM) rfhSP-A in the presence of Sytox Green. The change in the fluorescence of the dye was recorded as a function of time. The experiments were conducted at 37°C in PBS (pH 7.4). The results are the mean ± SD of three independent experiments, each in triplicate. A p-value < 0.001 was obtained for the one-way ANOVA followed by the Bonferroni multiple-comparison test: ***p < 0.001 compared to untreated bacteria (control).

We next assessed whether SP-A/LL-37 and rfhSP-A/LL-37 complexes affect the antimicrobial activity of LL-37 against *K. pneumoniae*, NTHi, and *P. aeruginosa.* These Gram-negative bacteria induce airway attacks in patients who are elderly, immunocompromised, or suffer from COPD, cystic fibrosis, or asthma (56–59). **Figure 7A** shows that when these Gram-negative bacteria were incubated with either SP-A or rfhSP-A and increasing concentrations of LL-37, neither SP-A nor rfhSP-A modifies the antibacterial activity of LL-37 against these three respiratory pathogens.

To assess the bacterial killing activity of SP-A/LL-37 and rfhSP-A/LL-37 complexes, we analyzed the membrane permeabilization of *K. pneumoniae* by using the fluorescent dye Sytox Green, whose fluorescence is enhanced upon binding to DNA once the bacterial cytoplasmic membrane is compromised (25). **Figure 7B** shows that LL-37 significantly increased the dye’s fluorescence at physiological salt concentrations, consistent with the bacterial membrane permeabilization properties of LL-37 (54). In line with bacterial killing assays (**Figure 7A**), bacteria treated with SP-A/LL-37 and rfhSP-A/LL-37 complexes showed Sytox Green permeabilization similar to those treated with LL-37 alone, confirming that SP-A/LL-37 and rfhSP-A/LL-37 complexes maintain LL-37 antimicrobial activity. Addition of SP-A or rfhSP-A alone did not affect the fluorescence of the dye, in line with previous reports (24, 25, 60).

**Figure 8:**
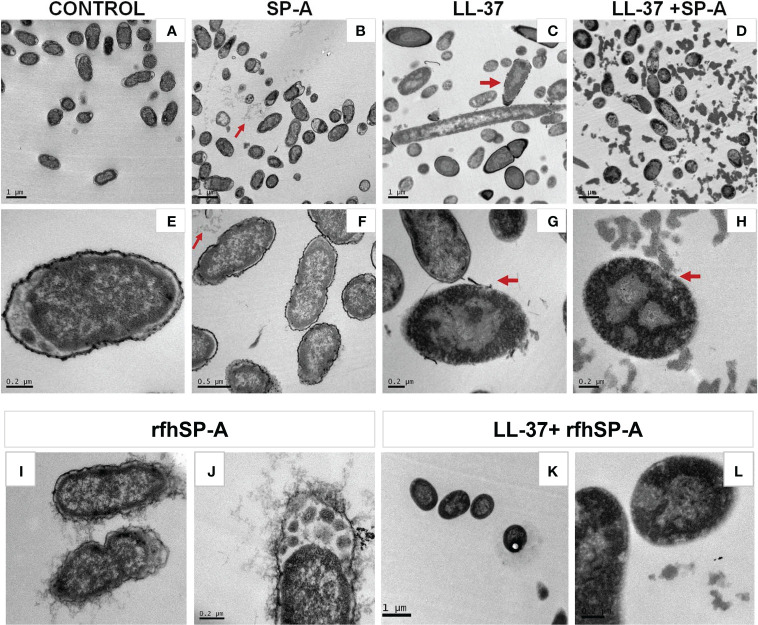
Effect of LL-37 and/or SP-A or rfhSP-A on the ultrastructure and morphology of *K. pneumoniae* K2. Untreated bacteria **(A, E)**, and bacteria treated with SP-A (0.3 µM) **(B, F)**, LL-37 (55.5 µM) **(C, G)**, LL-37+SP-A **(D, H)**, rfhSP-A (1.5 μM) **(I, J)**, and LL-37+rfhSP-A **(K, L)** for 30 min at 37°C are shown. Red arrows in B and F show self-aggregates of SP-A that do not interact with bacteria, while red arrows in **(C, G)** indicates disruption of the capsule and bacterial outer membrane by LL-37. Red arrows in **(D, H)** suggest the formation of protein-induced aggregates of outer membrane fragments. Representative micrographs of two independent experiments are shown.

To further characterize the effect of SP-A/LL-37 and rfhSP-A/LL-37 complexes on *K. pneumoniae* membranes, we recorded transmission electron microscopy (TEM) images of the bacteria in the absence and presence of LL-37, SP-A, rfhSP-A, SP-A/LL-37, and rfhSP-A/LL-37 complexes (**Figure 8**). LL-37 treatment caused several perturbations of the bacterial surface. The bacterial cell wall was fragmented, and dissociated fragments of the wall can be observed (**Figures 8C, G**, red arrows). In addition, **Figure 8G** shows that, intracellularly, LL-37 caused clustering of DNA (light grey) at the center of the cell, whereas ribosomes (dark grey) were directed towards the inner membrane. In contrast, untreated bacteria showed an even distribution of DNA and ribosomes (**Figure 8E**). These perturbations in *K. pneumoniae* induced by LL37 are similar to those observed in *E. coli* treated with LL-37 (61) or with chicken cathelicidin 2 (62). *K. pneumoniae* treated with SP-A/LL-37 and rfhSP-A/LL-37 complexes show damage identical to that of bacteria treated with LL-37 alone: disruption of bacterial wall and clustering of DNA in the center and ribosomes at the periphery of the cells (**Figures 8H, L** vs. **Figure 8G**). However, *K. pneumoniae* treatment with SP-A/LL-37 complexes results in a great number of large and dense aggregates throughout the preparation (**Figure 8D**), which were not observed in the presence of LL-37 alone (**Figure 8C** **)** or rfhSP-A/LL-37 complexes **(** **Figure 8K** **)**. These striking and abundant accumulations of small particulate matter could be formed by SP-A/LL-37-induced aggregation of bacterial outer membrane fragments, which would neutralize the inflammatory action of LPS.

As expected, SP-A alone did not cause any perturbation of the bacterial cell wall, and aggregates of SP-A, which do not interact with bacteria, can be observed in (**Figures 8B, F**) (red arrows). Curiously, these SP-A aggregates are not present in micrographs of bacteria treated with SP-A/LL-37 complexes (**Figures 8D, H**). Interestingly, rfhSP-A molecules were found associated with the bacterial surface (**Figures 8I, J**), confirming the binding of trimeric recombinant fragments of SP-A to *K. pneumonia* (60). This effect is attributed to its small size, which would allow the transit of rfhSP-A through the glycoconjugate structures of the capsule and the outer membrane. rfhSP-A exhibits small but significant direct microbicidal activity against *K. pneumoniae* by a mechanism that does not involve bacterial membrane permeabilization (60).

### 3.6 Effect of SP-A and rfhSP-A on LL-37-induced cell permeability

**Figure 9:**
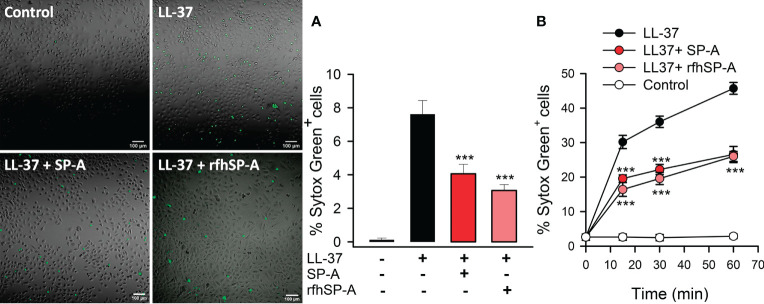
SP-A and rfhSP-A significantly reduce LL-37-induced uptake of Sytox Green by alveolar epithelial cells. Alveolar epithelial cells (A549 human cell line) were exposed to LL-37 (2.5 µM) in the presence or absence of SP-A (0.15 µM) or rfhSP-A (0.88 µM) for 30 min or the indicated times at 37°C. LL-37-induced Sytox Green (1 μM) incorporation into cells was analyzed by confocal microscopy **(A)** and flow cytometry **(B)**. In A, representative confocal micrographs and quantification of the % of Sytox Green+ cells are shown. Data are means ± S.E.M. of three independent experiments, with three biological replicates. For statistical analysis, ANOVA followed by Bonferroni multiple comparison test was used. ***p < 0.001, when cells treated with LL-37+SP-A or LL-37+rfhSP-A complexes are compared with cells treated with LL-37 alone.

LL-37 permeabilizes host cell membranes causing cytotoxicity (1, 5). Therefore, we next determined the effect of SP-A/LL-37 and rfhSP-A/LL-37 complexes on LL-37-induced host cell damage by measuring the incorporation of impermeable dyes in human cell lines. **Figure 9** shows that LL-37 induced Sytox Green incorporation in human pneumocytes, as shown by confocal microscopy (**Figure 9A**) and flow cytometry (**Figure 9B**). Notably, Sytox Green incorporation was significantly reduced after exposure of pneumocytes to SP-A/LL-37 and rfhSP-A/LL-37 complexes.

**Figure 10:**
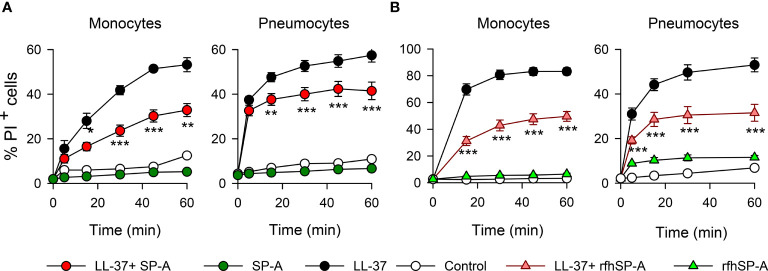
SP-A and rfhSP-A significantly reduce LL-37-induced uptake of propidium iodide by pneumocytes and monocytes. Kinetics of PI incorporation in human monocytes (U937) and pneumocytes (A549) were determined by flow cytometry. Cells were exposed to LL-37 (2.5 µM) in the presence and absence of **(A)** SP-A (0.15 µM) or **(B)** rfhSP-A (0.88 µM) at 37°C. Results are means ± S.E.M. of three different cell cultures, with three biological replicates. For statistical analysis, ANOVA followed by Bonferroni multiple comparison test was used. **p < 0.01, ***p < 0.001, when cells treated with LL-37+SP-A or LL-37+rfhSP-A complexes are compared with cells treated with LL-37 alone.

To confirm this protective role of SP-A and rfhSP-A, we evaluated the effect of LL-37, SP-A, rfhSP-A, SP-A/LL-37, and rfhSP-A/LL-37 complexes on propidium iodide **(**PI) uptake by human monocytes and pneumocytes by means of flow cytometry (**Figure 10**). LL-37 induced significant PI incorporation into human cells as a function of time, whereas SP-A and rfhSP-A alone did not. SP-A/LL-37 and rfhSP-A/LL-37 complexes significantly reduced the % of PI^+^ cells compared with those induced by LL-37 at any time of exposure. Taken together, our results show that SP-A/LL-37 and rfhSP-A/LL-37 complexes attenuate LL-37-induced cell permeability in human monocytes and pneumocytes.

**Figure 11:**
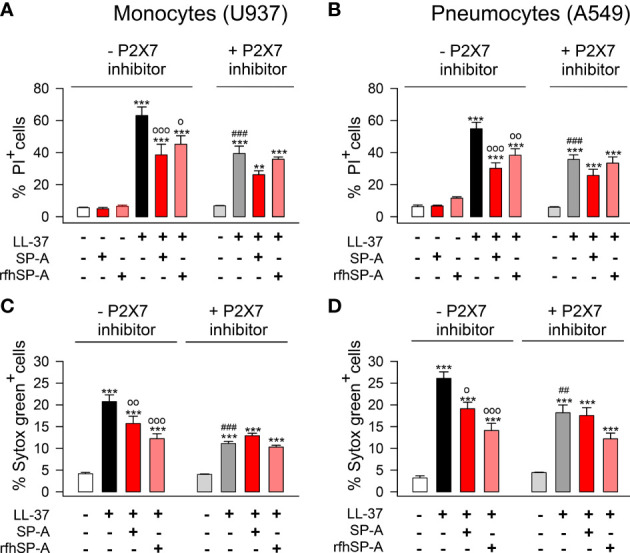
SP-A and rfhSP-A diminish the activation of P2X7 channels induced by LL-37 in pneumocytes and monocytes. **(A, C)** human U937 monocytes and **(B, D)** A549 pneumocytes were pre-treated with 10 µM A-438079, an inhibitor of the purinergic receptor P2X7, or with its vehicle (DMSO) for 15 min at 37°C. Then, cells were incubated with LL-37 (2.5 µM) in the presence or absence of SP-A (0.15 µM) and rfhSP-A (0.88 µM) for 1 hour. The entry of PI **(A, B)** or Sytox Green **(C, D)** to the cells was determined by flow cytometry. The P2X7 channel inhibitor partially reduced the entry of PI or Sytox Green induced by LL-37. Results are means ± S.E.M. of four different cell cultures, each with three biological replicates. For statistical analysis, ANOVA followed by Bonferroni multiple comparison test was used. **p < 0.01, ***p < 0.001 when LL-37-treated samples are compared with untreated controls. ^o^p < 0.05, ^oo^p < 0.01, and ^ooo^p < 0.001 when cells treated with SP-A/LL-37 or rfhSP-A/LL-37 complexes are compared with cells treated with LL-37. ^###^p < 0.01 when the effect of the P2X7 inhibitor is compared with the same sample without inhibitor.

Next, we explored whether LL-37-induced PI or Sytox Green uptake by human pneumocytes was mediated by P2X7 channel activation, as previously reported in fibroblasts and monocytes (63, 64). To that end, we inhibited P2X7 with A-438079 inhibitor and analyzed the incorporation of PI and Sytox Green in LL-37 stimulated and unstimulated human monocytes and pneumocytes, in the presence and absence of SP-A and rfhSP-A (**Figure 11**). As expected, LL-37 cytotoxicity appears to be partially mediated by activation of P2X7 channels in these cells. SP-A/LL-37 or rfhSP-A/LL-37 complexes significantly reduced LL-37 cytotoxicity in the absence but not in the presence of the P2X7 antagonist. These results suggest that LL-37 molecules integrated into the SP-A/LL-37 and rfhSP-A/LL-37 complexes are unable to activate P2X7 channels, which seems to result in the formation of a P2X7 macropore (65).

### 3.7 SP-A/LL-37 and rfhSP-A/LL-37 complexes reduce LL-37-induced IL-8 secretion that is dependent on P2X7 activation

**Figure 12:**
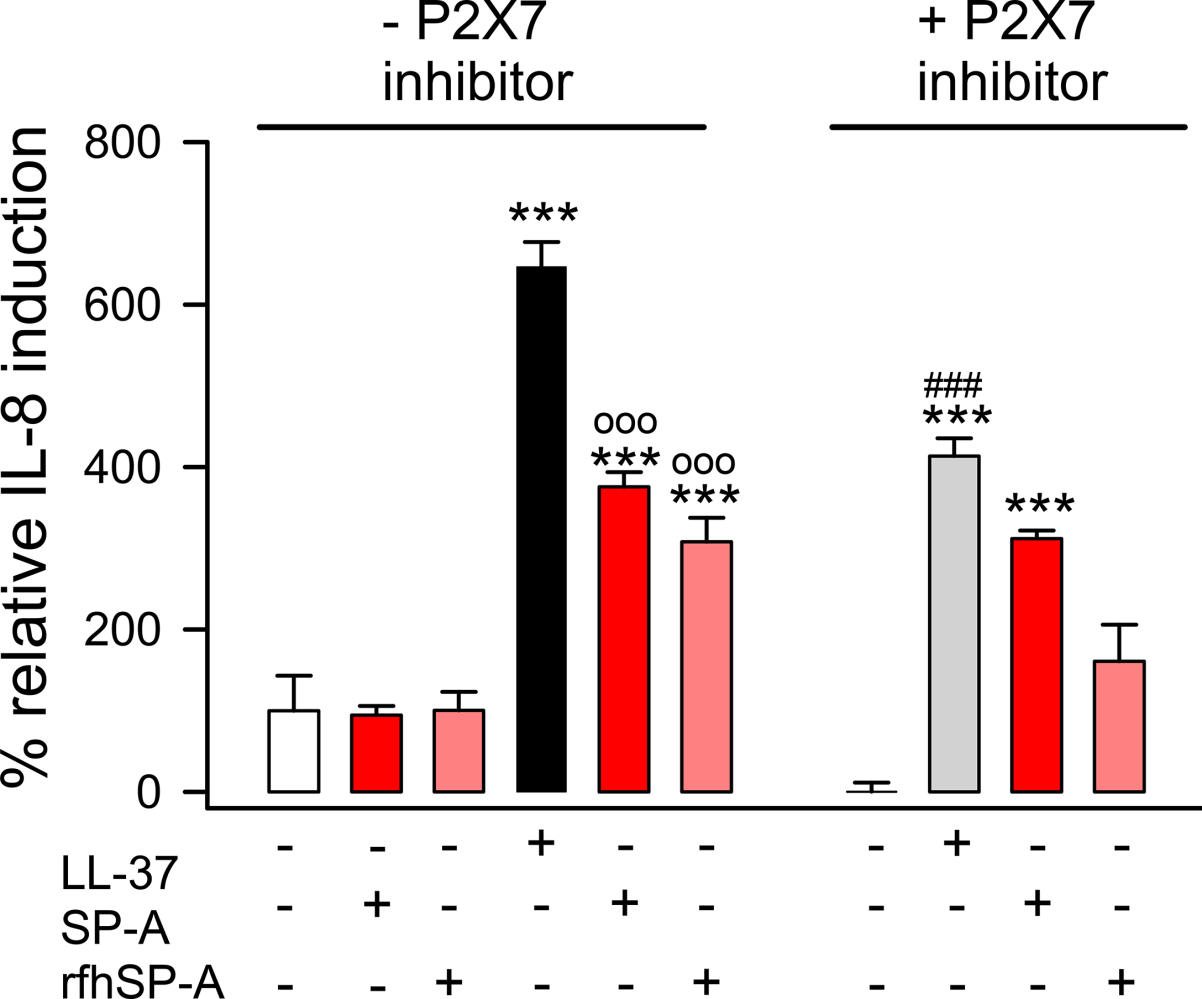
SP-A and rfhSP-A significantly reduce LL-37-induced secretion of IL-8 by alveolar epithelial cells. IL-8 release by human alveolar epithelial A549 cells was determined by ELISA from supernatants of cells pre-treated with and without an inhibitor of the purinergic receptor P2X7 (A-438079) or its vehicle (DMSO) for 15 min at 37°C. Then, cells were exposed to LL-37 (15 μM) in the presence and absence of SP-A (0.15 μM) or rfhSP-A (0.82 μM) for 24h at 37°C. Results are means ± S.E.M. of two different cell cultures with three biological replicates. For statistical analysis, ANOVA followed by Bonferroni multiple comparison test was used. ***p < 0.001 when LL-37-treated samples are compared with their respective untreated controls. ^ooo^p < 0.001 when cells treated with SP-A/LL-37 or rfhSP-A/LL-37 complexes are compared with cells treated with LL-37. ^###^p < 0.01 when the effect of the P2X7 inhibitor is compared with the same sample without inhibitor.

LL-37 enhances the release of chemoattractant cytokines such as IL-8 by alveolar epithelial cells (13, 14). Thus, we evaluated whether the interaction of SP-A or rfhSP-A with LL-37 could affect LL-37-induced IL-8 secretion by human alveolar epithelial cells, and whether this process was dependent on P2X7 activation as was previously reported on respiratory tract smooth muscle cells and mast cells (66, 67). **Figure 12** shows that LL-37 significantly induced IL-8 release by human pneumocytes, which was significantly reduced when LL-37 molecules remained integrated in SP-A/LL-37 and rfhSP-A/LL-37 complexes. Inhibition of P2X7 channels decreased LL-37-induced IL-8 secretion. The effect of SP-A and rfhSP-A on LL-37-induced IL-8 secretion was not observed in the presence of the P2X7 inhibitor, suggesting that SP-A/LL-37 and rfhSP-A/LL-37 complexes block activation of P2X7 channels by LL-37.

## 4 Discussion

**Figure 13:**
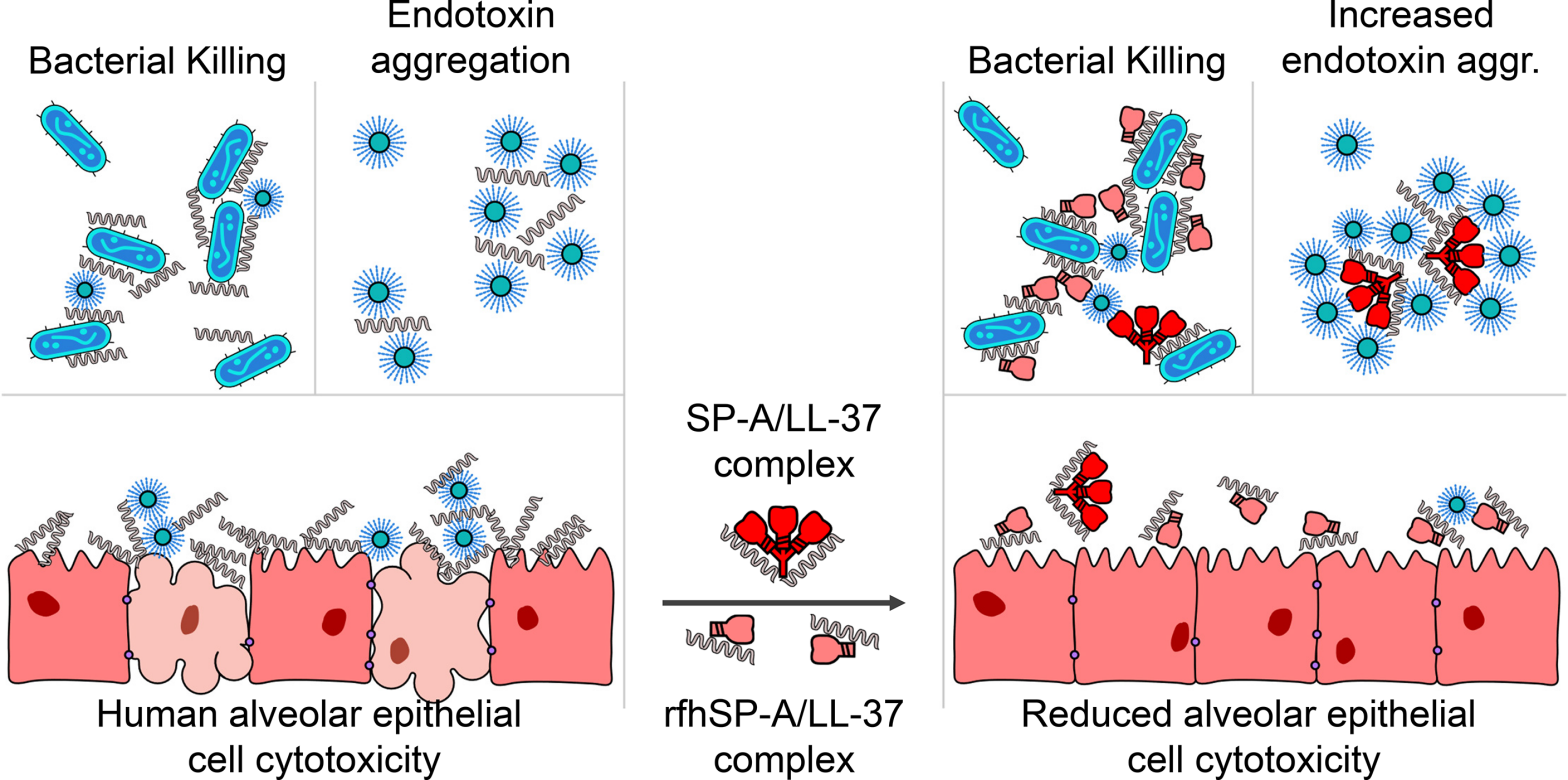
SP-A and rfhSP-A interact directly with LL-37. The LL-37 molecules integrated in the SP-A/LL-37 and rfhSP-A/LL-37 complexes are unable to induce membrane permeability in alveolar epithelial cells and the release of IL-8 by these cells, while retain or even enhance their antimicrobial properties against Gram-negative bacteria.

The collectin SP-A is secreted into the alveolar fluid, where it and other components of pulmonary surfactant protect the lung against alveolar collapse during the respiratory cycle. Additionally, SP-A protects the alveolar environment by facilitating pathogen clearance, limiting inflammation, and activating molecular and cellular mechanisms that help restore homeostasis (19–21, 24–27). On the other hand, LL-37 has low constitutive expression by epithelial and immune cells in the alveolus but is released into the alveolar fluid in large amounts in response to infection or injury (2, 3). High local concentrations of LL-37 can trigger tissue injury by binding to and permeabilizing host cell membranes. How alveolar cells are protected from LL-37 cytotoxicity is unknown. In this study we show that SP-A and LL-37 interact directly, and this interaction endows LL-37 with antimicrobial properties against Gram-negative respiratory pathogens while reducing its cytotoxic and inflammatory actions (**Figure 13**).

Direct binding between SP-A and LL-37 was assessed by tryptophan fluorescence of SP-A. LL-37 binds SP-A with high affinity in the absence (*K_D_* = 0.01 ± 0.007 pM) and presence (*K_D_* = 0.45 ± 0.006 nM) of physiological salt concentrations. This indicates the contribution of electrostatic and hydrophobic forces in the binding of LL-37 to SP-A. Similar *K_D_* values ​​were obtained for the interaction of LL-37 with rfhSP-A, a recombinant trimeric fragment of human SP-A which lacks the N-terminal domain and most of the collagen domain (**Figure 1**). This indicates that the binding of LL-37 to SP-A occurs *via* the globular domain or *via* the α-helical neck domain. The globular domains of SP-A recognize a wide repertoire of ligands, including surfactant phospholipids, bacterial lipids, carbohydrates, proteins, and nucleic acids (21, 44, 53, 55). Given that SP-A (or rfhSP-A) bound to LL-37 retained its ability to bind to LPS vesicles or phospholipid vesicles, it is possible that SP-A (or rfhSP-A) binds LL-37 by a non-globular domain, probably *via* the α-helical domain. The neck domain between the collagen domain and the globular domains forms an α-helical coil structure (68). The amino acid sequence of this region is characterized by an «a-b-c-d-e-f-g-d» heptad repeat pattern, where residues «a» and «d» are hydrophobic amino acids. The α-helical coiled coil structure is one of the essential domains for trimerization and is also involved in SP-A binding to the leucine-rich portion of CD14 (69).

Binding of LL-37 to SP-A or rfhSP-A resulted in the formation of molecular complexes as revealed by circular dichroism and dynamic light scattering experiments. When the SP-A/LL-37 and rfhSP-A/LL-37 complexes interacted with lipid vesicles that mimic the inner and outer membranes of Gram-negative bacteria, the α-helix content of the SP-A/LL-37 and rfhSP-A/LL-37 complexes was critically increased as did LL-37 alone. Since native LL-37 requires an α-helical structure for antibacterial activity (46), these results suggest that the helical content of SP-A/LL-37 and rfhSP-A/LL-37 complexes might be sufficient to retain antibacterial activity against Gram-negative bacteria. Consistently, we found that when *K. pneumoniae* K2, nontypable *H. influenzae* (NTHi), and *P. aeruginosa* were incubated with increasing concentrations of LL-37 in the presence and absence of SP-A (at concentrations within the ranges found in human lung fluid) (70), the formation of SP-A/LL-37 complexes did not modify the bactericidal activity of LL-37 against these three respiratory pathogens. Similar results were obtained for rfhSP-A/LL-37 complexes. The pathogens used are commonly isolated from sputum specimens of patients with lower respiratory tract infections, which are among the most common infectious diseases affecting humans. NTHi is a non-capsulated Gram-negative bacterium that exacerbates COPD (56) and colonizes the lower respiratory tract of patients with neutrophilic asthma (57). *P. aeruginosa* preferentially colonizes immunocompromised patients and is commonly associated with cystic fibrosis patients (58). *K. pneumoniae* causes serious nosocomial infections and is associated with high rates of mortality, particularly in immunocompromised patients or patients with mechanical ventilation (59).

Retention of LL-37 bactericidal activity after binding to SP-A or rfhSP-A was confirmed by Sytox Green bacterial permeabilization assay and visualized by transmission electron microscopy. Interestingly, treatment of *K. pneumoniae* with SP-A/LL-37 complexes resulted in many dense aggregates visible by TEM, which were not observed in the presence of LL-37 alone. We suggest that this accumulation of small particles could be formed by SP-A/LL-37-induced aggregation of LPS-containing bacterial outer membrane fragments. Indeed, here we show that the ability of SP-A/LL-37 complexes to aggregate LPS vesicles was significantly greater than that of LL-37 alone. Aggregation of LPS particles reduces LPS toxicity (71) and facilitates its phagocytosis by alveolar macrophages (72). LPS interaction with its receptor complex is blocked by LPS aggregation induced by SP-A (55) and LL-37 (73), which reduces proinflammatory cytokine production. The neutralization of LPS to control the inflammatory response induced by endotoxins may be as important as the cytotoxic effect on Gram-negative bacteria. Furthermore, LPS promotes the destabilization and alteration of the biophysical activity of pulmonary surfactant (74). Both SP-A and LL-37 can act as scavengers of LPS, protecting pulmonary surfactant from the inhibitory effects of LPS.

Relevantly, our results demonstrated that the formation of SP-A/LL-37 and rfhSP-A/LL-37 complexes prevents LL-37 cytotoxicity in host cells. LL-37 has only moderate selectivity for bacterial lipids and can cause membrane permeabilization of host cells at concentrations of LL-37 not much higher than those that cause bacterial death (54). The mechanism by which SP-A and its recombinant fragment reduce LL-37-induced membrane permeability in pneumocytes and monocytes, without affecting LL-37-induced killing of pathogenic bacteria, is unknown.

The outer layer of eukaryotic cell membranes is composed primarily of zwitterionic phosphatidylcholine (PC) and sphingomyelin (SM), phospholipids that do not electrostatically attract cationic antimicrobial peptides. This suggests the involvement of hydrophobic interactions between positively charged LL-37 and zwitterionic phospholipids. Since the N-terminus of LL-37 is hydrophobic, it is possible that a cluster of N-terminal regions of oligomerized LL-37 may initiate host cell membrane binding (75). Consistently, a peptide truncated at the N-terminal region of LL-37 (FF-33) is less able to penetrate PC membranes and has less hemolytic activity (75). However, FF-33 and LL-37 has identical antimicrobial activity (75). Indeed, the core fragment of LL-37 (residues 17–32) is considered to encompass the major antimicrobial region of LL-37 (76). It is tempting to speculate that binding of SP-A or rfhSP-A to LL-37 might hide the N-terminal hydrophobic region of LL-37, diminishing its effect on host cell membranes. However, further studies are required to address this issue.

LL-37-induced permeability in host cells appears to be mediated in part by activation of P2X7 purinergic channels, as determined in monocytes (47, 63) and fibroblasts (64) and confirmed in this study on pneumocytes. It has been proposed that the sustained activation of P2X7 channels by LL-37 gives rise to a large non-selective macropore that allows uptake of molecules up to 900 Da (65), such as the fluorescent dyes used in this study. The mechanism through which LL-37 activates the P2X7 macropore is unknown, and the direct interaction between LL-37 and P2X7 has not been studied. It has been proposed that LL-37 may activate P2X7 channels by modifying lateral membrane organization and membrane fluidity (65) since P2X7 channel activity is predominantly inhibited by cholesterol through direct interactions with the transmembrane domain of P2X7 (77). Our results indicate that the presence of SP-A or rfhSP-A significantly reduces LL-37-induced membrane permeability to Sytox Green and PI in pneumocytes and monocytes, as well as IL-8 release by pneumocytes compared to cells stimulated with LL-37 alone. We found that both processes depended on the activation of the P2X7 channels by LL-37, and that LL-37 molecules integrated in the SP-A/LL-37 and rfhSP-A/LL-37 complexes seem unable to activate these channels.

Our results show that SP-A offers protection in the lung against the cytotoxic activities of LL-37 similar to plasma apolipoprotein A-I blockage of LL-37 at cytotoxic concentrations for endothelial cells (14, 16, 17). Binding of LL-37 to SP-A might have several advantages in addition to protection against LL-37 cytotoxicity on host cell membranes. The alveolar fluid of normal lungs contains a substantial excess of SP-A, which is found around epithelial and immune cells and pulmonary surfactant membranes (21, 44, 53). The tight binding of SP-A to surfactant membranes allows for its strategic location at the air-liquid interface, which is the first line of defense against inhaled pathogens and endotoxins entering the alveoli (21, 44, 53). Here we show that the binding of LL-37 to native SP-A does not interfere with the Ca^2+^-dependent phenomena by which SP-A self-associates and induces the aggregation of surfactant bilayers, increasing cohesion among the surfactant membranes and facilitating surfactant biophysical activity (44, 53). LL-37 binding to SP-A could serve to collect and kill inhaled microbes at the air-liquid interface. SP-A, which binds LL-37 and other antimicrobial peptides, together with surfactant membranes, containing SP-B and SP-C, seems to function simultaneously as the primary antimicrobial defense at the air-liquid interface and as a protective layer against alveolar collapse.

In patients with COPD, cystic fibrosis, and sarcoidosis, the concentrations of SP-A and surfactant lipids are decreased (78–80), while the concentration of LL-37 is elevated (4, 81). This can lead to an exacerbation of these diseases since the proinflammatory response is amplified. The involvement of the P2X7 receptor in the pathogenesis of pulmonary emphysema and COPD is well established (82–85). Reduced levels of SP-A are due to reduced synthesis caused by epithelial cell injury and increased degradation caused by protease release of neutrophils (86) or pathogens (87). In this study we show that a trimeric recombinant fragment of SP-A is sufficient to neutralize the cytotoxic and proinflammatory effects of cathelicidin in alveolar epithelial cells, without affecting the microbicidal activity of LL-37 against *K. pneumoniae*, nontypeable *H. influenzae*, and *P. aeruginosa*, which can exacerbate COPD, asthma, and lung fibrosis and induce airway attacks in immunocompromised patients and the aged (88). These results suggest that rfhSP-A could be used therapeutically in chronic respiratory diseases characterized by an increase of LL-37. Future studies will address the therapeutic benefits of rfhSP-A.

## Data availability statement

The raw data supporting the conclusions of this article will be made available by the authors, without undue reservation.

## Author contributions

Conceptualization: LT, BG-F, NK, JJ, and CC; methodology: LT, BG-F, and NK; investigation: all authors; formal analysis: all authors; writing—original draft preparation: LT; writing—review and editing: CC; supervision: CC; project administration: CC; funding acquisition for this study: JJ and CC. All authors contributed to the article and approved the submitted version.

## Funding

This research was funded by the Spanish Ministry of Science and Innovation through Grants RTI2018-094355‐B‐I00 and PID2021-123044OB-I00 to CC and by the Swedish Research Council (2020–02434) to JJ. LT was the recipient of a contract (PEJ-2020-AI/BMD-17865) from the Consejería de Educación, Juventud y Deporte of Comunidad de Madrid (Spain) and the European Social Funding Program.

## Acknowledgments

We thank the ICTS Electron Microscopy National Center and the Confocal Microscopy Unit of the Complutense University of Madrid for their excellent technical support Reference.

## Conflict of interest

The authors declare that the research was conducted in the absence of any commercial or financial relationships that could be construed as a potential conflict of interest.

## Publisher’s note

All claims expressed in this article are solely those of the authors and do not necessarily represent those of their affiliated organizations, or those of the publisher, the editors and the reviewers. Any product that may be evaluated in this article, or claim that may be made by its manufacturer, is not guaranteed or endorsed by the publisher.
