# Supplementary material for: The collectin SP-A and its trimeric recombinant fragment protect alveolar epithelial cells from the cytotoxic and proinflammatory effects of human cathelicidin in vitro
Source: Front Immunol. 2022 Aug 29;13:994328. doi: 10.3389/fimmu.2022.994328 (PMC9464622; doi:10.3389/fimmu.2022.994328)
Supplement: Supplementary file 1 [file DataSheet_1.pdf]

Supplementary Material

The collectin SP-A and its trimeric recombinant fragment protect alveolar epithelial cells from the cytotoxic and proinflammatory effects of human cathelicidin *in vitro*

Lidia de Tapia[1], Belén García-Fojeda[1], Nina Kronqvist[2], Jan Johansson[2], and Cristina Casals[1]*

[1] Department of Biochemistry and Molecular Biology, Complutense University of Madrid, Madrid, Spain.

[2] Department of Biosciences and Nutrition, Neo, Karolinska Institutet, Huddinge, Sweden.

Corresponding Author
Cristina Casals.
Department of Biochemistry and Molecular Biology, Faculty of Biology, Complutense University of Madrid, 28040 Madrid, Spain,
Tel.: (34) 913944261,
ccasalsc@ucm.es

Keywords: Cathelicidin LL-37, collectin SP-A, trimeric recombinant fragment, antimicrobial activity, cytotoxicity, inflammation, P2X7 channel, alveolar epithelial cells.

Running Title: SP-A protects from LL-37 cytotoxicity

Number of words: 8610 (Main text)

Number of Figures: 13 (and 3 supplementary figures).

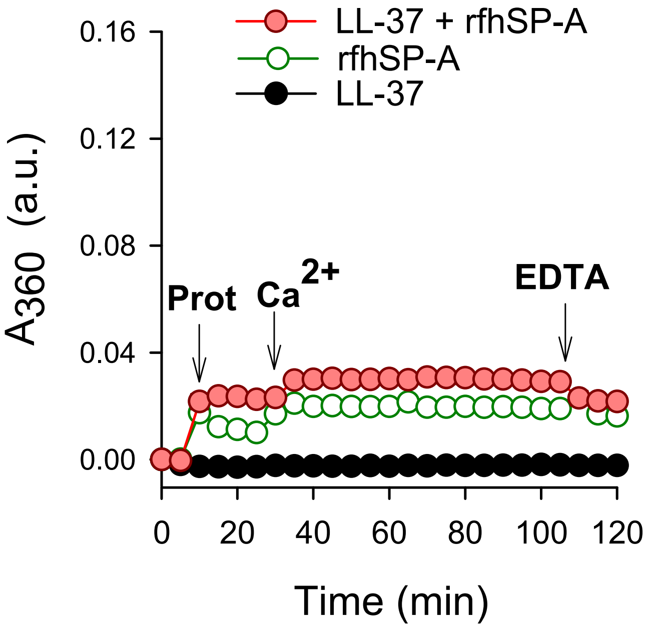

Figure S1. rfhSP-A (0.45 µM), which lacks the N-terminal domain and the collagen domain, could not undergo Ca^{2+}-dependent self-aggregation either in the presence or absence of LL-37 (1.1 µM). Results are shown as means ± SD of three independent experiments.

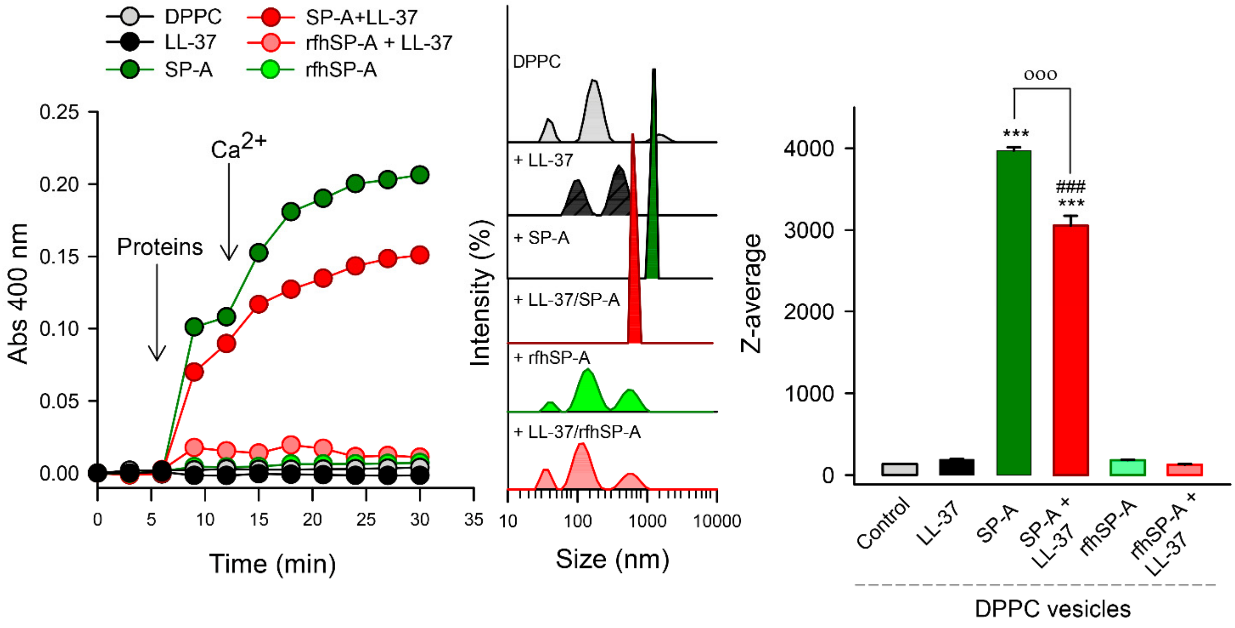

Figure S2. Ca^{2+}-dependent DPPC vesicle aggregation induced by SP-A, LL-37, rfhSP-A, and combinations thereof. DPPC vesicles (50 µg/ml) in 5 mM Tris-HCl buffer, pH 7.4, containing 150 mM NaCl and 0.1 mM EDTA were used. The left panel represent one representative experiment of three DPPC vesicle aggregation induced by proteins measured by turbidity at 400 nm. The central panel shows the samples used for turbidity measurements analyzed by DLS. The right panel shows the Z-average measurements of these samples. Results are the mean ± SD of three experiments. For statistical analysis, ANOVA followed by Bonferroni multiple comparison test was used. *** p<0.001 when protein-treated samples are compared with the control (DPPC vesicles). ^{OOO} p<0.001 when samples with SP-A are compared with SP-A/LL-37 samples. $^{\#\#\#}$ p<0.01 when SP-A/LL-37 and LL-37 samples are compared. The concentrations of proteins were 75 nM SP-A, 1.1 µM LL-37, and 0.43 µM rfhSP-A. The concentration of Ca^{2+} was 2.5 mM.

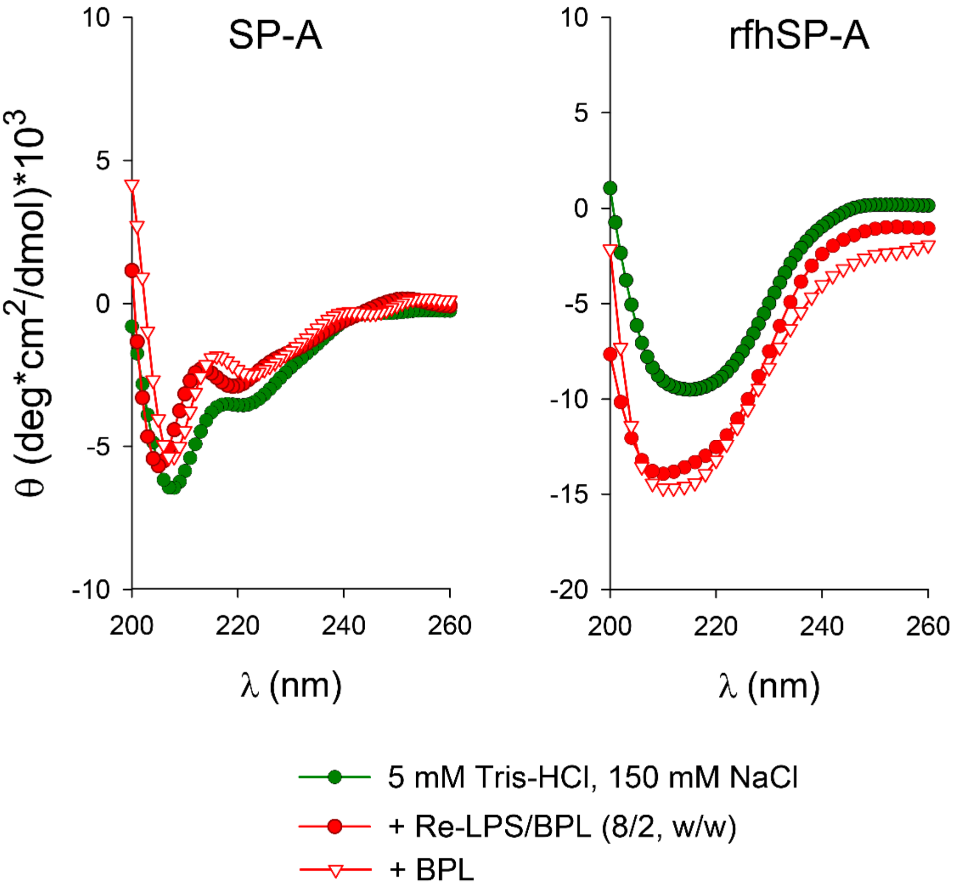

Figure S3. Secondary structure of SP-A and rfhSP-A in the presence and absence of bacterial membranes. Changes in the molar ellipticity of SP-A and rfhSP-A in the absence (green circles) and presence of lipid vesicles mimicking the outer membrane (red circles) composed of Re-LPS/BPL (8:2, w/w) (1 mg/ml) and inner membrane (open triangles) (BPL: POPE/POPG/CL, 67/23/10, w/w/w)(1 mg/ml) of Gram-negative bacteria. Measurements were performed at 25 °C in 5mM Tris-HCl, 150mM NaCl buffer, pH 7.4. Protein concentrations were 75 µg/ml for SP-A, and rfhSP-A. A representative experiment of three are shown.
